# Supplementary material for: Integration of alternative fragmentation techniques into standard LC-MS workflows using a single deep learning model enhances proteome coverage
Source: Nat Methods. 2026 Mar 23;23(4):805–14. doi: 10.1038/s41592-026-03042-9 (PMC13076210; doi:10.1038/s41592-026-03042-9)
Supplement: Supplementary file 1 — Supplementary Notes, Supplementary Table 1, Supplementary Figs. S1–S25. [file 41592_2026_3042_MOESM1_ESM.pdf]

nature methods

Supplementary information

https://doi.org/10.1038/s41592-026-03042-9

Integration of alternative fragmentation techniques into standard LC-MS workflows using a single deep learning model enhances proteome coverage

In the format provided by the authors and unedited

Table of Contents

Supplementary Notes **2**

Optimisation of DC gradients for ion transfer within the Omnitrap 2

Duty cycle of irradiation of precursor ions by free electrons in the Omnitrap 2

Discussion and interpretation of fragment-ion annotation results 2

Acquisition and Peculiarities of the ETciD data 3

Negative control test of the MultFrag deep learning model 4

Evaluation of train/test split 4

Contribution of prediction of retention time on the overall performance of the model 6

Enzyme-specific fragmentation patterns 6

Applicability of the deep learning model to other datasets 8

Supplementary references **9**

Supplementary Table 1. Definitions of fragment ion types **11**

Supplementary Figure S1. Breakdown of the contribution of each enzyme to the total numbers of PSMs **12**

Supplementary Figure S2. Density contour plots of hyperscore distributions against molecular weight per charge state **13**

Supplementary Figure S3. Density contour plots of hyperscore distributions against *m/z* values per charge state **14**

Supplementary Figure S4. Density contour plots of hyperscore distributions against *m/z* values per charge state (by *vs* abcxyz) **15**

Supplementary Figure S5. Frequencies and log ion proportions in ETciD data **16**

Supplementary Figure S6. Log ion proportions of ions annotated in ECD data **17**

Supplementary Figure S7. Log ion proportions of ions annotated in EID data **18**

Supplementary Figure S8. Log ion proportions of ions annotated in HCD data **19**

Supplementary Figure S9. Log ion proportions of ions annotated in UVPD data **20**

Supplementary Figure S10. Benchmarking the ECD, EID and UVPD datasets against the Multifrag and HCD 2020 Prosit models **21**

Supplementary Figure S11. Oktoberfest rescoring separates target from decoy (ECD) **22**

Supplementary Figure S12. Oktoberfest rescoring separates target from decoy (EID) **23**

Supplementary Figure S13. Oktoberfest rescoring separates target from decoy (HCD) **24**

Supplementary Figure S14. Oktoberfest rescoring separates target from decoy (UVPD) **25**

Supplementary Figure S15. Oktoberfest rescoring separates target from decoy (ETciD) **26**

Supplementary Figure S16. Distributions of target PSMs in ECD are charge-separated after rescoring in Oktoberfest **27**

Supplementary Figure S17. Gain/share/loss at the peptide level (Oktoberfest vs MSFragger) **28**

Supplementary Figure S18. Gain/share/loss (Oktoberfest vs MSFragger) in the ETciD data **29**

Supplementary Figure S19. Identification of estimated true positives at different FDR thresholds per enzyme **30**

Supplementary Figure S20. Performance of ETciD in DDA searches **31**

Supplementary Figure S21. Mean normalised intensities of fragments in doubly charged precursors of 12-amino-acid-long peptides **32**

Supplementary Figure S22. Mean normalised intensities of fragments in triply charged precursors of 12-amino-acid-long peptides **33**

Supplementary Figure S23. Sequence logos of all 12-amino-acid-long peptides **34**

Supplementary Figure S24. Sequence logos of unique 12-amino-acid-long peptides **35**

Supplementary Figure S25. Rescoring of the 3+ precursors from the publicly available ETD chymotrypsin dataset (Ref. [77]) **36**

Supplementary notes

Optimisation of DC gradients for ion transfer within the Omnitrap

For the characterisation of UVPD, we constructed an experiment using short 30 min LC gradients and used a tryptic cell lysate digest as the analyte. There are two parameters of UVPD that can be changed, namely, the number and energy of laser pulses, and we began with varying the number of laser pulses at a fixed energy of 6 mJ/pulse. In the analysis of the data acquired in UVPD experiments, we started with using only *b* and *y* ions for identification, as they were previously shown to be the most abundant in UVPD of tryptic peptides [61, 62, 63] (see Supplementary Table 1 for structures and definitions of all fragment ions considered in this work). The analysis shows that the numbers of identified peptide-spectrum matches (PSMs) and peptides reaches a maximum at two pulses and quickly starts to fall beyond that point (Extended Data Fig. 1c). As key characteristic of UVPD is the formation of *a,x,c,z* (in addition to *b* and *y*) ions, we asked the question if the trend was the same when considering these fragment types. The numbers of identifications indeed did follow the same trend but were remarkably lower than those obtained using *b* and *y* fragments (Extended Data Fig. 1d). Based on the earlier reports [61, 62, 63], we expected a higher proportion of identifications using *a,x,c,z* in our data. As *b* and *y* fragments can also be generated through 'background' collisional fragmentation which can occur in multiple locations during ion transfer, we investigated the origins of these ions. We repeated the experiment but now without triggering the UV laser and observed a significant population of *b* and *y* ions. We found that the ion transfer to and within the Omnitrap was the source and so we tested several new approaches. In the original approach, ions would be transferred from the HCD cell of Exploris through the transfer hexapoles and into the Q2 segment of the Omnitrap (Extended Data Fig. 1e). Subsequently, the ions would be transferred into the Q8 segment for UVPD using an 8V DC gradient between Q2 and Q8. We tried two other designs of ion transfer for UVPD: a) use of a 4V gradient in the last ion transfer step and b) injection into the Q5 segments and then transfer to Q8 using a 4V DC gradient (Extended Data Fig. 1e). Comparing the numbers of PSMs, peptides, and proteins obtained we found that the third design allows reduction of the number of PSMs sequenced by the factor of 4 to negligible levels (Extended Data Fig. 1f). We therefore switched to this design and continued the general optimisation (see the article).

Duty cycle of irradiation of precursor ions by free electrons in the Omnitrap

In a typical Omnitrap ExD experiment, precursor ions are transferred into the reaction chamber and undergo irradiation by electrons emitted by a heated filament during a specified amount of time [64]. The efficient electron irradiation time, however, is only half of the time that the ions spend in the reaction chamber. This is due to the rectangular waveform potentials applied to the electrodes of the Omnitrap that alternate with radiofrequency (RF) [64] (Extended Data Fig. 1g). These potentials allow electrons to enter the ion trap only during the positive RF phase and repel otherwise (Extended Data Fig. 1h). We provide total ion confinement times for ExD throughout this paper, and the efficient irradiation times can be obtained by dividing these values by two.

Discussion and interpretation of fragment-ion annotation results

For all LCMS datasets, we performed an automated annotation of major fragment types expected in EID, ECD, and UVPD (Supplementary Table 1) in the Oktoberfest platform. After gathering annotation statistics over the entire dataset, we could establish an output ion fragment dictionary, based on the prevalence of any ion we searched for. This varies heavily between the

fragmentation methods. For instance, while HCD is known to produce primarily *a*, *b* and *y* fragments, EID and UVPD, on average, contain appreciable proportions of *a*, *a+1*, *b*, *c*, *x*, *x+1*, *y*, *z*, and *z+1* (Fig. 3a). More detailed distributions of ion proportions per enzyme are given in Extended Data Fig. 3 and Supplementary Fig. S5-9. Notably, the frequencies of these ions are very similar in EID and UVPD, with the former having on average more *c*, *x+1*, and *z*, and the latter having more *y* fragments. While *a+1* signal may in principle correspond to the ^{13}C isotope of an *a* ion, the comparison of [*a+1*]/[*a*] ratio in HCD, EID, and UVPD suggests that a good proportion of *a+1* in EID and UVPD spectra are rather ions originating from gas-phase electron- and photon-based chemistries. The formation of *a+1* fragments in UVPD have been described in multiple publications [65], whereas in EID they have been largely ignored and to the best of our knowledge were only reported by Ly *et al*. [66] In ECD, radical *a+1* ions are believed to form *via* solvation of charge located at the backbone nitrogen (as opposed to solvation of charge at carbonyl required for the generation of *c*/*z* ions) [67]. In our data, they comprise on average 5% of all annotated signals which is almost the same proportion as in HCD. The frequency of *a* ions in ECD is however negligible (potentially random matches) as opposed to 11% in HCD (Fig. 3a), which confirms the non-collisional nature of *a+1* in ECD. The high abundance of *z+1* ions in ECD can be explained by the presence of ^{13}C isotopes of *z* fragments, but in this case we would expect the [*z+1*]/[*z*] ratio to be much lower at 35-50%, similar to [*a+1*]/[*a*] ratio in HCD. This suggests that *z+1* ions are possibly products of hydrogen atom transfer (HAT). The *c-1* fragments in ECD are far less frequent than *z+1* ions that they are believed to complement, the phenomenon also observed in ETD [68, 69]. Although its causes have not been discussed, a potential explanation can be given by reduced stability of N-centred radicals (assumed for *c-1* ions) [70], as compared to relatively stable C-centred radicals found in *a+1*, *x+1*, and *z* fragments [71]. Remarkably, *c-1* ions are not present in UVPD and EID data, and *z+1* fragments are very likely to be ^{13}C isotopes of *z*, based on the [*z+1*]/[*z*] ratio (Fig. 3a, Extended Data Fig. 3). This suggests either non-ECD-like ion chemistry of their formation, or different energetics of the process reducing the propensity for HAT. Furthermore, *x* ions were previously reported to be by far the least abundant in UVPD of cationic peptides, hardly surpassing their frequency in HCD data (where they supposedly represent random matches) for different proteases [61]. Our data shows similar trend and suggests similar frequencies of *x* and *x+1* in UVPD. EID contains the highest proportions of *x* and *x+1* fragments among all fragmentation methods, with the frequency of *x+1* even higher than that of *x* (Fig. 3a, Extended Data Fig. 3). Finally, HCD, EID, and UVPD are dominated by *b* and *y* fragments. Interestingly though, while the frequency of *b* ions is uniform across these three techniques and comprises on average approximately 25%, the average frequency of *y* ions tends to increase from the relatively modest 26% in EID through 35% in UVPD to nearly one half in HCD (Fig. 3a, Extended Data Fig. 3), indicating different mechanisms may be involved. In ECD, *b* and *y* ions are relatively scarce and originate either from collisions with the buffer gas (*i.e.*, background CID) or, in the case of *y* ions, potentially from solvation of charge located at the backbone nitrogen as discussed above.

Acquisition and Peculiarities of the ETciD data

The peptide dataset that we are presenting in this paper was acquired on two instruments. We collected ECD, EID, HCD and UVPD datasets on the Exploris-Omnitrap hybrid instrument, and the ETciD data were acquired on an Ascend Tribrid mass spectrometer. The mechanisms and products of ETciD and ECD are very similar, and therefore it is tempting to draw a performance comparison between the two datasets. However, there are several features of our ETciD data that discourage us from performing such comparisons. First, for technical reasons, the ETciD dataset

was acquired with approximately 5% fewer fractions than each of the Omnitrap datasets. Second, we observed an issue with the scan acquisition on the Ascend Tribrid, which we believe may be associated with a bug in the acquisition soft- or firm-ware. The bug results in compromised quality of the first MS2 scan immediately after an MS1 survey scan which reduces the number of high-quality MS2 scans by approximately 5% and may affect other scans too. This seems to be a common problem on at least Ascend instruments, as a very similar issue has been reported recently by Riley and co-workers [72]. Finally, the low-mass cutoff settings were marginally different between the Omnitrap and Ascend data which will lead to the general intensity profile of the fragmentation spectra to be distinct.

Negative control test of the MultFrag deep learning model

We sought to make sure that our new model does not "hallucinate", *i.e.* it doesn't produce suspiciously good predictions where it is not expected to. For example, ECD and HCD have completely orthogonal fragmentation mechanisms and products, except for the low possibility of ECD-generated *y*-ions having some overlap with HCD-generated *y*-ions in fragmentation patterns. We therefore expect Pearson correlation coefficients (PCC) between HCD model predictions and ECD data to be either anticorrelated or be very weakly correlated. Supplementary Figure S10a indeed confirms this assumption, showing a good portion of predictions by the "Prosit_2020_HCD" model [73] for trypsin ECD data being evenly spread between 0.0 (no correlation) to 1.0 (high correlation). Moreover, a collection of PSMs are clustered at -1.0 PCC which corresponds to spectra that contain only peaks that cannot be explained by the HCD prediction model, *i.e.* they contain exclusively *c*- and *z*-ions but no *b*- or *y*-type ions. We also asked the question, how HCD predictions correlate with intensities in EID and UVPD data. Supplementary Figure S10b-c shows that "Prosit_2020_HCD" predictions for trypsin EID and UVPD data have largely positive PCCs, although spanning to much lower values compared to the predictions made by the new "Multifrag" EID and UVPD models. This is not surprising considering that both EID and UVPD produce relatively abundant *b* and *y* ions, but the weaker correlations with HCD predictions points at the presence of additional fragment series and a potentially different, non-CID, mechanisms of the *b*- and *y*-ion formation.

Evaluation of train/test split

For our published model we elected to split train/val/test spectra randomly. Although the dataset is deduplicated into unique modified sequence/charge/fragmentation method instances, this still could lead to data leakage with respect to unmodified sequences, which is the more commonly used method of splitting PSM data. In order to assuage concerns of a test set that gives an overly optimistic evaluation of model performance because of duplicate sequences in both train and test sets, we separated the results by unique unmodified sequences.

Table SN1: Comparison of models trained with random train/val/test splits and split on unmodified sequence.

	Median in-train sequences PCC<br>Random split	Median out-of-train sequences PCC<br>Random split
HCD	0.946	0.917
UVPD	0.927	0.882

EID	0.888	0.789
ECD	0.917	0.868
Overall	0.913	0.875

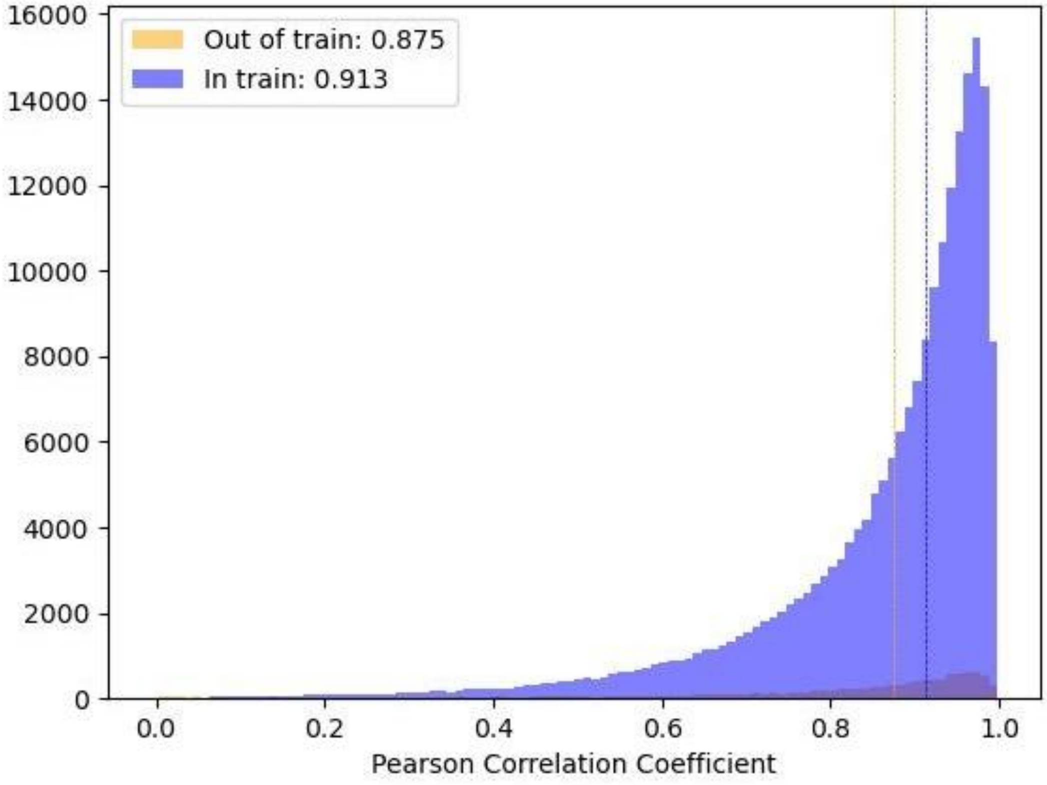

Figure SN1. Histograms of Pearson Correlation Coefficients (PCC) in the test set, separated by unique unmodified peptide sequences, which do not occur in our training set, and leaked sequences that do. The median PCCs are denoted by the vertical dashed lines, as well as their values displayed in the legend.

We observe that the difference in median PCC scores is modest, comparing 0.913 for leaked sequences to 0.875 for unique sequences. The 4% disparity in prediction accuracy for unseen sequences indicates that the model's effectiveness does drop somewhat when encountering new data. We note that this is unlikely to have substantial consequences in application, *e.g.* rescoring, where many other features are often considered alongside PCC. This is evident in the similarity of the rescoring results for the *Arabidopsis Thaliana* DIA data to the *Human* DIA data even though *A. Thaliana* has very low sequence overlap with the human data used to train the model. Similarly, in the original Prosit work on CID [73] substantial improvements for chymotrypsin data were observed upon rescoring even though the model was built exclusively on trypsin data. Furthermore, when the model was trained on chymotryptic data, the rescoring results displayed negligible changes.

Contribution of prediction of retention time on the overall performance of the model

The modest rescoring gains in ECD data (Fig. 4c, Supplementary Figure S17) prompted us to investigate how much the prediction of fragment intensities contribute to the improvements in the ECD data along with prediction of retention times (RT) of peptides. We used the rescoring of the trypsin ECD data by the "Prosit_2020_HCD" model [73] without prediction of RT as a baseline, as such rescoring is expected to produce only minor improvements (see Supplementary Note "Negative control test of the MultiFrag deep learning model"). Indeed, as shown in Extended Data Figure 6a, such rescoring has little effect with the number of gains hardly surpassing the number of losses. When the same model was used for rescoring, this time with prediction of RT turned on (Extended Data Figure 6b), the effect was much more pronounced, with the number of gains of approximately 16k PSMs. Interestingly, a nearly identical result was obtained when the new "Prosit_2025_Multifrag" model was used for rescoring without RT predictions (Extended Data Figure 6c), indicating that at least for the trypsin ECD data the value of RT and intensities predictions have nearly the same magnitude. Finally, when both RT and fragment intensities were predicted at the same time, the number of gains edged a bit higher than when each of these aspects was predicted separately (Extended Data Figure 6d).

Enzyme-specific fragmentation patterns

We investigated the effect of charge state and choice of enzyme on the fragmentation patterns for each activation technique. Protease choice affects the number and location of basic and acidic residues impacting formation and stability of proton-directed fragment ions. Aromatic and amino acids containing heteroatoms influence radical-initiated fragments. Each enzyme creates distinct patterns with respect to acidic, basic and aromatic residues. The results of rescoring using spectral libraries generated by our deep learning model (Figure 4 and Supplementary Figures S17, S18) suggest that such variation causes search engines trouble to correctly identify non-tryptic peptide spectra. For example, the main beneficiary of rescoring using the original CID- [73] and the new "Multifrag" (Fig. 3) Prosit deep learning models is chymotrypsin which is known for creating peptides with 'orthogonal' physicochemical properties when compared to trypsin (Extended Data Figure 2a). Considering the well-known case of CID of chymotryptic peptides there is an expectation that the *b* and *y* ion populations will be affected, yet our data suggests the fragmentation model applied by the modern search engine used in this work struggles to effectively capture these changes. We investigated if we could discern any general enzyme and activation method spectral patterns. Holistically, our data (Extended Data Figure 3, SI Figures S5-9) already showed that the frequencies of *b*,*y* types of ions in HCD, EID and UVPD are heavily shifted towards C-terminal *y* ions in trypsin and LysC data due to the presence of basic arginine and lysine residues at C termini. The effect is the opposite in LysN data where peptides contain basic lysine residues at N termini, and intermediate in GluC and chymotrypsin data where distribution of basic residues across peptide backbones is more or less random. Similarly, the frequency of *c* ions in ECD and ETciD is very much favoured in LysN data compared to trypsin and LysC.

We, furthermore, asked the question if our data would allow us to extract the general appearance of a spectrum for each enzyme and fragmentation context. To reduce complexity, we chose to focus on one length of peptide for all conditions. We chose 12-amino-acid long peptides (charge states 2 and 3), as they were the most frequently identified peptide length (no doubt related to our mass spectrometric acquisition parameters), see Extended Data Figure 2a. We plotted mean

normalised intensities of a_k, b_k, c_k, $x_{(12-k)}$, $y_{(12-k)}$, $z_{(12-k)}$ fragment ions for each dissociation position *k*=1,2,...,11 (Supplementary Figures S21 and S22). Pleasingly, we recreated a textbook doubly charged tryptic peptide HCD spectrum which is dominated by a *y*-ion series and a weaker *b*-ion series that is more prominent in the low *m/z* range. LysC HCD peptides were similar while LysN HCD spectra had the *b*-ion series dominate, although the *y*-ion series remained prominent across the whole spectrum. Both GluC and chymotrypsin produced similar average 2+ spectra where both *b* and *y* ions had almost equal prominence across the spectrum (Supplementary Figures S21). Triply charged spectra followed similar trends although *y*-ion series were always in the ascendancy. All these trends can be rationalised with the mobile proton fragmentation framework. Strikingly, the observed distributions reveal similarities between ECD and ETciD. In particular, the distributions of *c* and *z* types of fragments in ECD and ETciD follow nearly identical trends. Both show simple clean spectra for doubly charge tryptic peptides. The *z*-ion series dominates with a few prominent large *c* ions. The pattern for LysC is similar while LysN has a very dominant *c*-series and a weak low-mass *z* population. The spectra for GluC and chymotrypsin are somewhere in between LysC and LysN, although *c*-ion series are always dominating the high mass range and *z* ions the low mass range. ECD of 2+ precursors generally possess slightly more *z* ions than ETciD. Remarkably, the proportions of singly and doubly charged fragments are nearly the same in ECD and ETciD of 3+ precursors, Supplementary Figure S22.

The main difference between enzymes is the strength of the terminal fragments that are the most dominant in chymotryptic spectra. The distributions of *b* and *y* ions in EID and UVPD look very similar although different to HCD, potentially indicating similar mechanisms of their formation in UVPD and EID. EID, however, has substantially higher proportions of *x* ions compared to any other technique, and overall, the relative intensities of *a*, *c*, *x* and *z* ions compared to *b* and *y* fragments are higher in EID than in UVPD. Choice of enzyme seems to have less of an effect for EID compared to HCD, although *b* and *y* ions always remain prominent, and which series dominates follows the same pattern as HCD for each enzyme. Both *b* and *y* ion series are also prominent throughout all enzymatic conditions for UVPD although the dominance of each series follows more strongly (than EID) the trends in HCD. This observation serves as evidence to the earlier hypothesis put forward by Julian [74] suggesting that the reaction of UVPD may proceed via either direct dissociation following photon-induced electron excitation into a dissociative orbital, or "CID-like" fragmentation following internal conversion of the photon energy into vibrational modes. Triply charged peptides for both EID and UVPD contain multiple prominent ion series; however, it does appear that terminal fragments (C-terminal for trypsin and LysC and N-terminal for the rest) are incredibly dominant which is not as apparent when looking at individual spectra suggesting consistency of formation rather than a dominant fragmentation pathway.

The generalised trends for fragmentation spectra suggested that there might also be trends within the successfully sequenced peptides. As a second approach we generated sequence logos reflecting occurrence frequencies of amino acids across the 12-amino-acid long peptides although we removed the enzyme specific terminal residues for clarity (Supplementary Figures S23 and S24). In general, the logos were very similar across the board. Glutamates and leucines were the dominant residues with alanines and serines being prominent. Enzyme-specific logos reflected specificity, *e.g.* Glu-C lacked glutamates and chymotrypsin had a diminished leucine population. The subsets of peptides unique to each fragmentation technique have slightly different sequence logos. For example, ECD favours peptides with arginine and lysine residues scattered across the backbone, apparently due to their affinity to a proton affecting ECD kinetics. UVPD and EID have slightly higher propensities to peptides containing phenylalanine and

tyrosine residues, due to their ability to absorb a photon or stabilise a radical. The lack of a clear logos supports the premise that all techniques have excellent relatively bias-free sequencing efficiencies once we can correctly predict and score spectra.

Applicability of the deep learning model to other datasets

In order to determine the quality and generalisability of the generated deep learning model, it is necessary to benchmark it against an external dataset. In this regard, one limitation entailing straight from the very motivation for this work, is lack of any such ECD, EID or UVPD datasets to benchmark against. To gauge the generalisability of our data we chose to address the question through the common mechanism for two of our fragmentation datasets. Electron capture dissociation was initially discovered by Zubarev and McLafferty [75] in the late 90s and was shown to be an excellent fragmentation technique for proteins and heralded top down proteomics. The use of free electrons made it difficult to implement on traditional proteomics instruments. Syka, Coon and Hunt developed [76] electron transfer dissociation (ETD), an approach that allowed implementation of electrons on regular 'trapping' instruments and generated similar results to ECD. A large body of work has demonstrated that the two techniques employ the same underlying mechanisms and produce similar data when parameters are appropriately controlled. Now our own data (Supplementary Figures S21, S22) has shown that two techniques are very similar at the peptide level. The results indicate that not only are ECD and ETD close but that our model will be able to rationalise other implementations of both ETD and ECD. It is noteworthy this similarity is observed despite the data being generated with quite different approaches and acquired on completely different platforms (ETD in a Thermo Linear Ion Trap vs ECD in the Omnitrap – both recorded spectra in Orbitraps). In other words, the fundamental mechanisms dominate over the implementation, and rescoring using our model would lead to improved identification rates. We decided to go one step further and analysed a public ETD dataset. We chose the recent Nature Biotechnology paper by Coon and co-workers [77]. Unfortunately, this public dataset (and pretty much all ETD datasets) recorded the spectra in the linear ion trap creating difficulty in performing a robust comparison. A secondary issue was that the authors chose to maximise the complementarity of HCD, EThcD and ETD by choosing EThcD and ETD to focus on the 200-800 m/z range and for ETD to be applied only to 3+ and higher charge states. The authors set the low-mass cut off for the ETD data to 120 Th, far lower than in our acquisitions which is set by the instrument (at higher values) due to our focus on the typical mass range 400-1500 m/z. Still this dataset is the most comprehensive dataset available and acquired on a modern Tribrid instrument. When comparing our model to the data (charge state 3+) we obtained a median value of 0.7 (Supplementary Figure S25a), which was pleasing considering the large disparity in acquisition parameters. Inspecting spectra across a range of Pearson values (Extended Data Fig. 10) against the predicted ETciD spectrum and our acquired ETciD and ECD spectra, we noted a couple of trends. The first is the similarity between ETciD predicted, ETciD experimental and ECD experimental spectra. The second is, the Coon dataset spectra contain the expected predicted fragments; however, the fragment intensities are clearly skewed towards the lower mass range relative to our data. Furthermore, fragments are present in the low-mass range which are not predicted by our model or observed in our experimental data. These discrepancies can be explained by the choice of the low-mass cut off, an underappreciated parameter that can have a dramatic effect on the MS profile. Although there is a fragment mass range intensity profile skew and the data are acquired on low resolution and low mass accuracy instrument, we asked the question if rescoring using our model would have any benefit. We observed a net gain of 1.2% at the PSM level and 2.4% at the peptide level while also achieving a

substantially superior separation of target and decoy results (Supplementary Figure S25b-d). Although the gains are modest, the actual predictions clearly were beneficial. The fragmentation ions were correctly predicted where data overlapped.

Supplementary references

[59] Biemann K. Appendix 5. Nomenclature for peptide fragment ions (positive ions). *Methods Enzymol.* 193:886-7 (1990).

[60] Chu, I. K. *et al.* Proposed nomenclature for peptide ion fragmentation. *International Journal of Mass Spectrometry* **15**, 24-27 (2015).

[61] Greer, S. M., Parker, W. R. & Brodbelt, J. S. Impact of Protease on Ultraviolet Photodissociation Mass Spectrometry for Bottom-up Proteomics. *Journal of Proteome Research* **14**, 2626-2632 (2015).

[62] Fort, K. L. *et al.* Implementation of Ultraviolet Photodissociation on a Benchtop Q Exactive Mass Spectrometer and Its Application to Phosphoproteomics. *Analytical Chemistry* **88**, 2303-2310 (2016).

[63] Kolbowski, L., Belsom, A. & Rappsilber, J. Ultraviolet Photodissociation of Tryptic Peptide Backbones at 213 nm. *Journal of the American Society for Mass Spectrometry* **31**, 1282-1290 (2020).

[64] Papanastasiou, D. *et al.* The Omnitrap Platform: A Versatile Segmented Linear Ion Trap for Multidimensional Multiple-Stage Tandem Mass Spectrometry. *Journal of the American Society for Mass Spectrometry* **33**, 1990-2007 (2022).

[65] Brodbelt, J. S., Morrison, L. J. & Santos, I. Ultraviolet Photodissociation Mass Spectrometry for Analysis of Biological Molecules. *Chemical Reviews* **120**, 3328-3380 (2020).

[66] Ly, T., Yin, S., Loo, J. A. & Julian, R. R. Electron-induced dissociation of protonated peptides yields backbone fragmentation consistent with a hydrogen-deficient radical. *Rapid Communications in Mass Spectrometry* **23**, 2099-2101 (2009).

[67] Zubarev, R. A., Good, D. M., Savitski, M. M. Radical a-Ions in Electron Capture Dissociation: On the Origin of Species. *Journal of The American Society for Mass Spectrometry* **23**, 1015-1018 (2012).

[68] Chalkley, R. J., Medzihradszky, K. F., Lynn, A. J., Baker, P. R. & Burlingame, A. L. Statistical Analysis of Peptide Electron Transfer Dissociation Fragmentation Mass Spectrometry. *Analytical Chemistry* **82**, 579-584 (2010).

[69] Peters-Clarke, T. M., Riley, N. M., Westphall, M. S. & Coon, J. J. Practical Effects of Intramolecular Hydrogen Rearrangement in Electron Transfer Dissociation-Based Proteomics. *Journal of the American Society for Mass Spectrometry* **33**, 100-110 (2022).

[70] Hioe, J., Šakić, D., Vrček, V. & Zipse, H. The stability of nitrogen-centered radicals. *Organic & Biomolecular Chemistry* **13**, 157-169 (2015).

[71] Hioe, J. & Zipse, H. Radical stability and its role in synthesis and catalysis. *Organic & Biomolecular Chemistry* **8**, 3609-3617 (2010).

[72] Veth T, Kothlow K, Riley N. Understanding m/z range settings for MS/MS scans: a case study with intact glycopeptides. ChemRxiv. 2025; doi:10.26434/chemrxiv-2025-6wsqc. This content is a preprint and has not been peer-reviewed.

[73] Gessulat, S., Schmidt, T., Zolg, D.P. *et al*. Prosit: proteome-wide prediction of peptide tandem mass spectra by deep learning. *Nat Methods* **16**, 509–518 (2019).

[74] Ryan R. Julian, The Mechanism Behind Top-Down UVPD Experiments: Making Sense of Apparent Contradictions, *J. Am. Soc. Mass Spectrom.,* **28** (9), 1823-1826 (2017).

[75] Roman A. Zubarev, Neil L. Kelleher, and Fred W. McLafferty. Electron Capture Dissociation of Multiply Charged Protein Cations. A Nonergodic Process. *J Am Chem Soc* **120** (13), 3265-3266 (1998).

[76] J.E.P. Syka,J.J. Coon,M.J. Schroeder,J. Shabanowitz, and D.F. Hunt. Peptide and protein sequence analysis by electron transfer dissociation mass spectrometry, *Proc. Natl. Acad. Sci. U.S.A.* **101** (26) 9528-9533 (2004).

[77] Sinitcyn, P., Richards, A.L., Weatheritt, R.J. *et al*. Global detection of human variants and isoforms by deep proteome sequencing. *Nat Biotechnol* **41**, 1776–1786 (2023).

Supplementary Table 1. Definitions of fragment ion types. Nomenclatures of singly-charged fragment ions proposed by Biemann (Ref [59], used in this work) and Chu *et al.* (Ref [60]) and corresponding elemental compositions of fragments. R, R' and R'' stand for arbitrary amino acid side chains. An unpaired electron is marked as "•".

ion type		elemental composition	ion type		elemental composition
Ref [59]	Ref [60]		Ref [59]	Ref [60]	
a	$[a]^+$	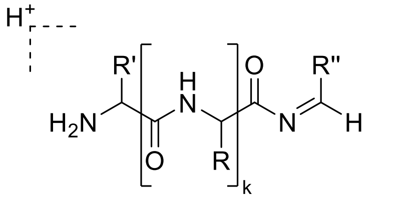	a+1	$[a+H]^{•+}$	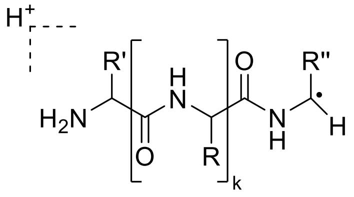
b	$[b]^+$	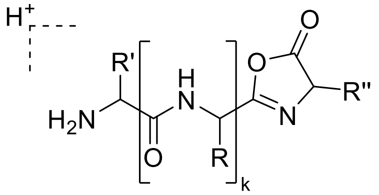			
c	$[c+2H]^+$	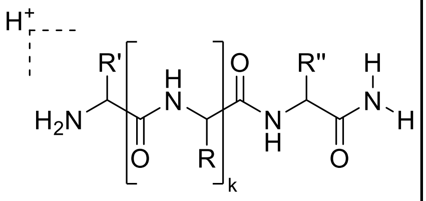	c-1	$[c+H]^{•+}$	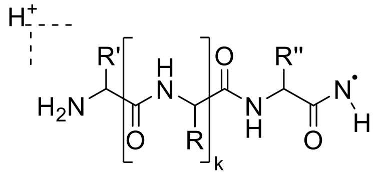
x	$[x]^+$	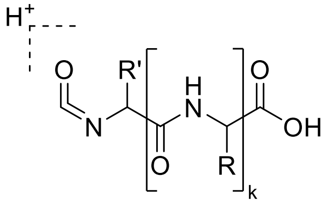	x+1	$[x+H]^{•+}$	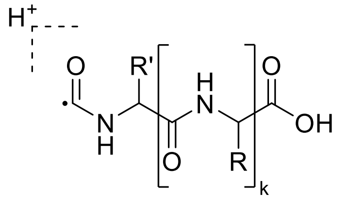
y	$[y+2H]^+$	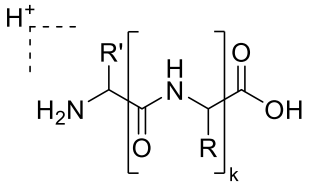			
z	$[z+H]^{•+}$	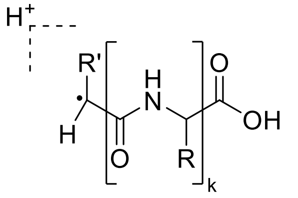	z+1	$[z+2H]^+$	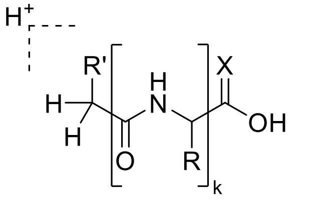

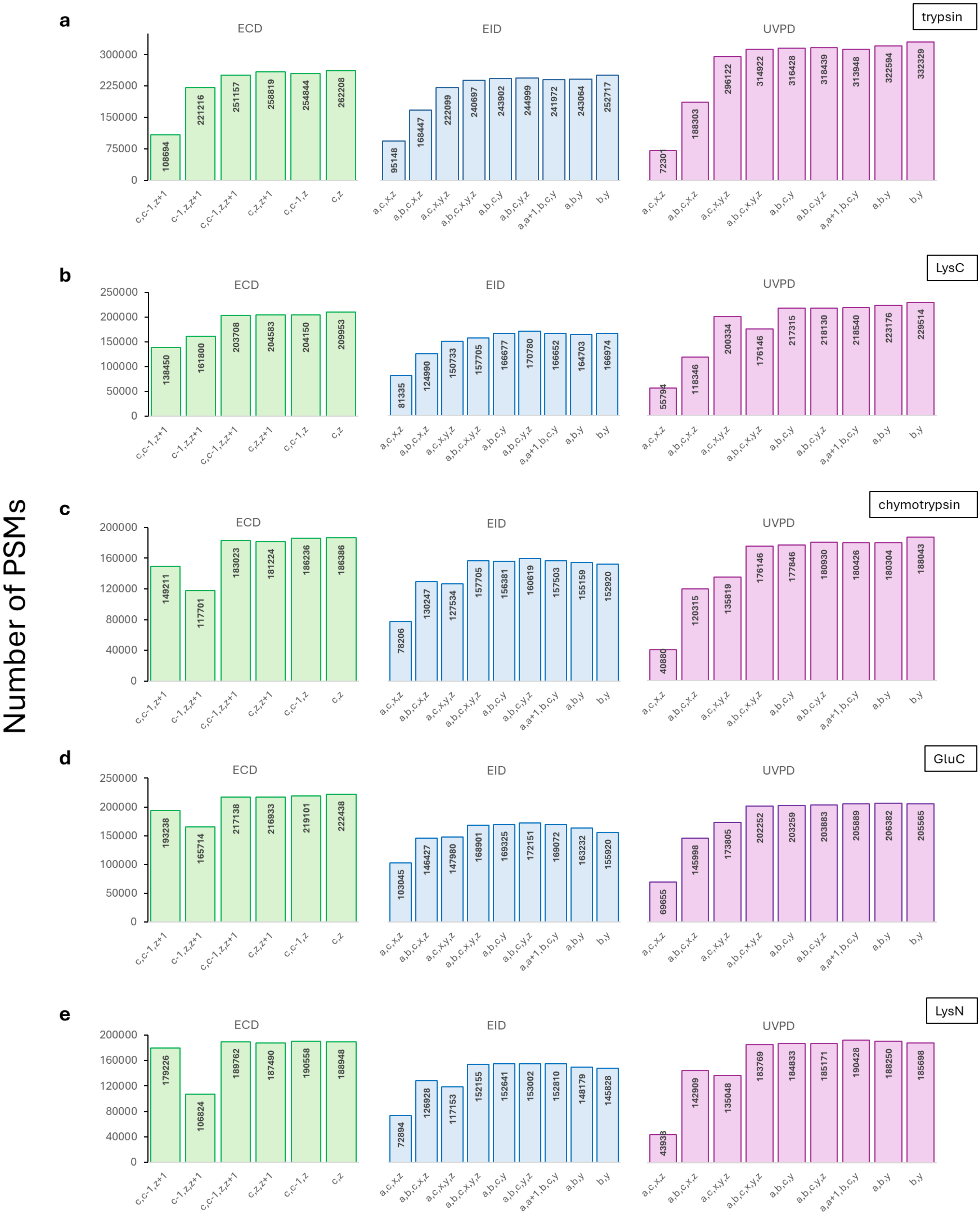

Supplementary Figure S1. Breakdown of the contribution of each enzyme to the total numbers of PSMs. Numbers of peptide-spectrum matches (PSMs) in ECD, EID, and UVPD experiments identified using different combinations of fragment types in trypsin (a), LysC (b), chymotrypsin (c), GluC (d), and LysN (e) datasets.

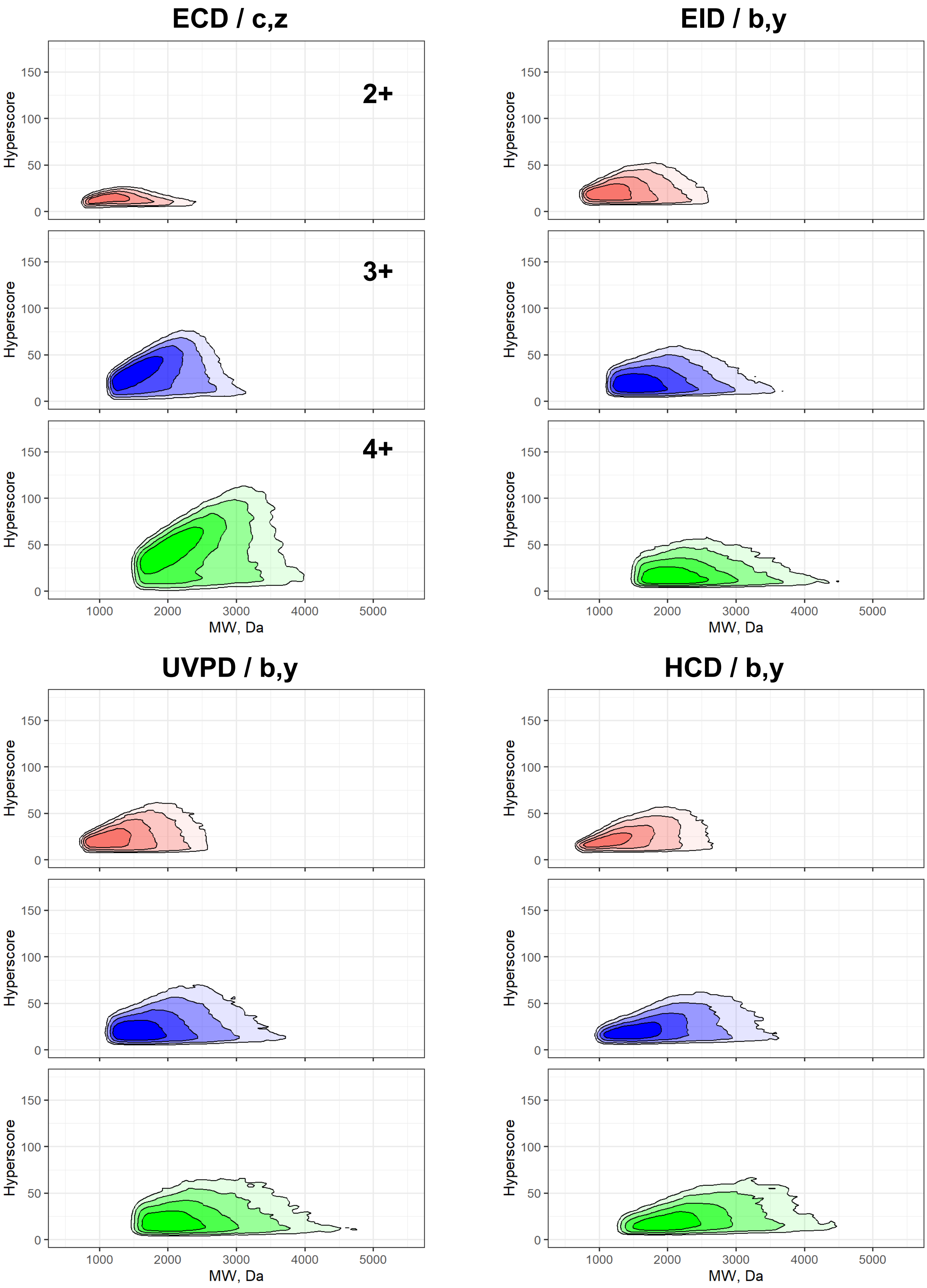

Supplementary Figure S2. Density contour plots of hyperscore distributions against molecular weight per charge state. Density contour plots of hyperscore distributions of 2+ (red), 3+ (blue), and 4+ (green) charge states of unique PSMs (unique combination of amino-acid sequence, charge, and modification selected by highest hyperscore) plotted against molecular weights (MW) of precursors acquired in ECD using *c* and *z* types of fragments and in EID, UVPD and HCD using *b* and *y* types of fragments. Contour lines demarcate the smallest regions to contain 50, 80, 95 and 99% of data points.

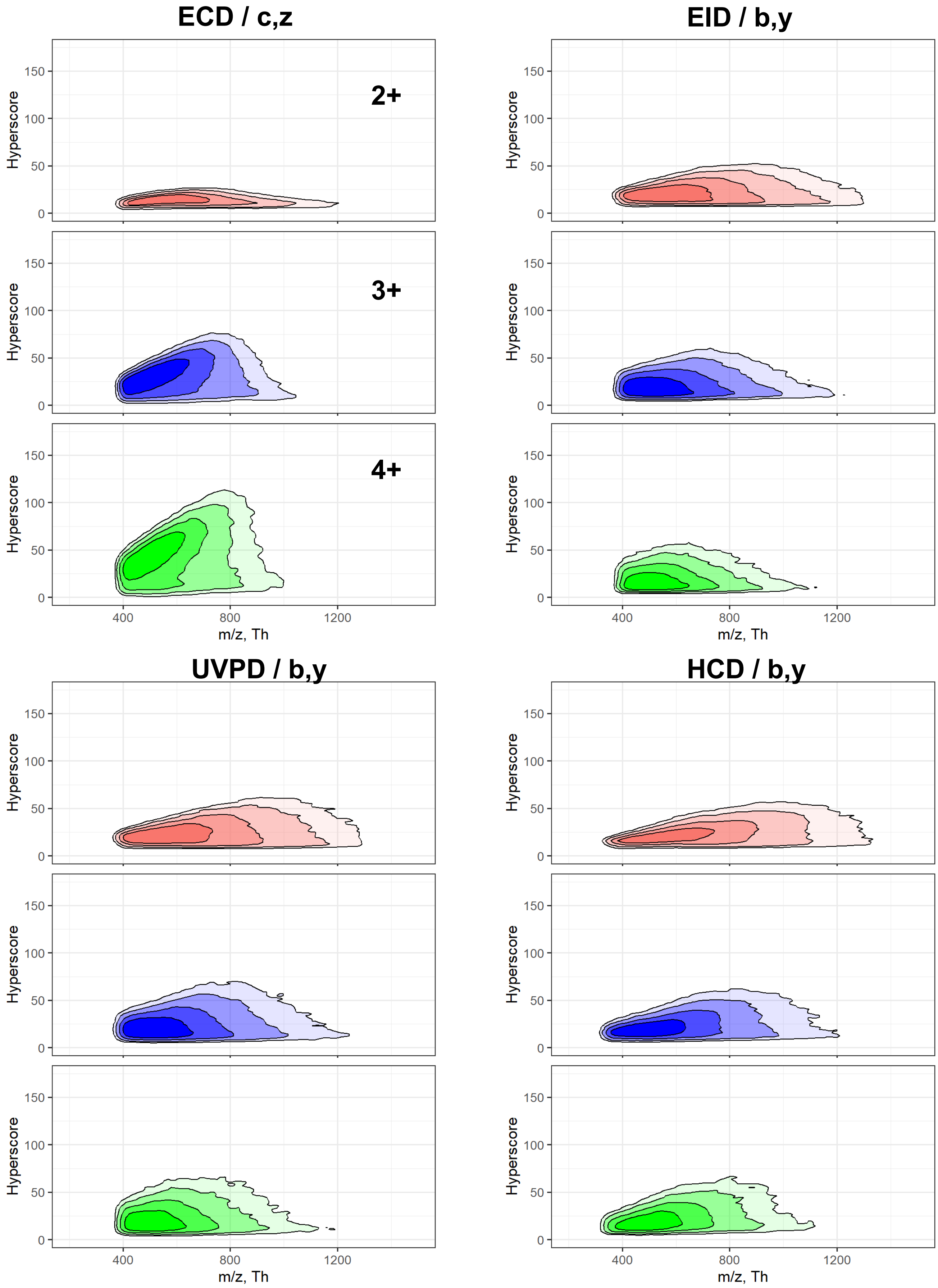

Supplementary Figure S3. Density contour plots of hyperscore distributions against *m/z* values per charge state. Density contour plots of hyperscore distributions of 2+ (red), 3+ (blue), and 4+ (green) charge states of unique PSMs (unique combination of amino-acid sequence, charge, and modification selected by highest hyperscore) plotted against *m/z* values of precursors acquired in ECD using *c* and *z* types of fragments and in EID, UVPD and HCD using *b* and *y* types of fragments. Contour lines demarcate the smallest regions to contain 50, 80, 95 and 99% of data points.

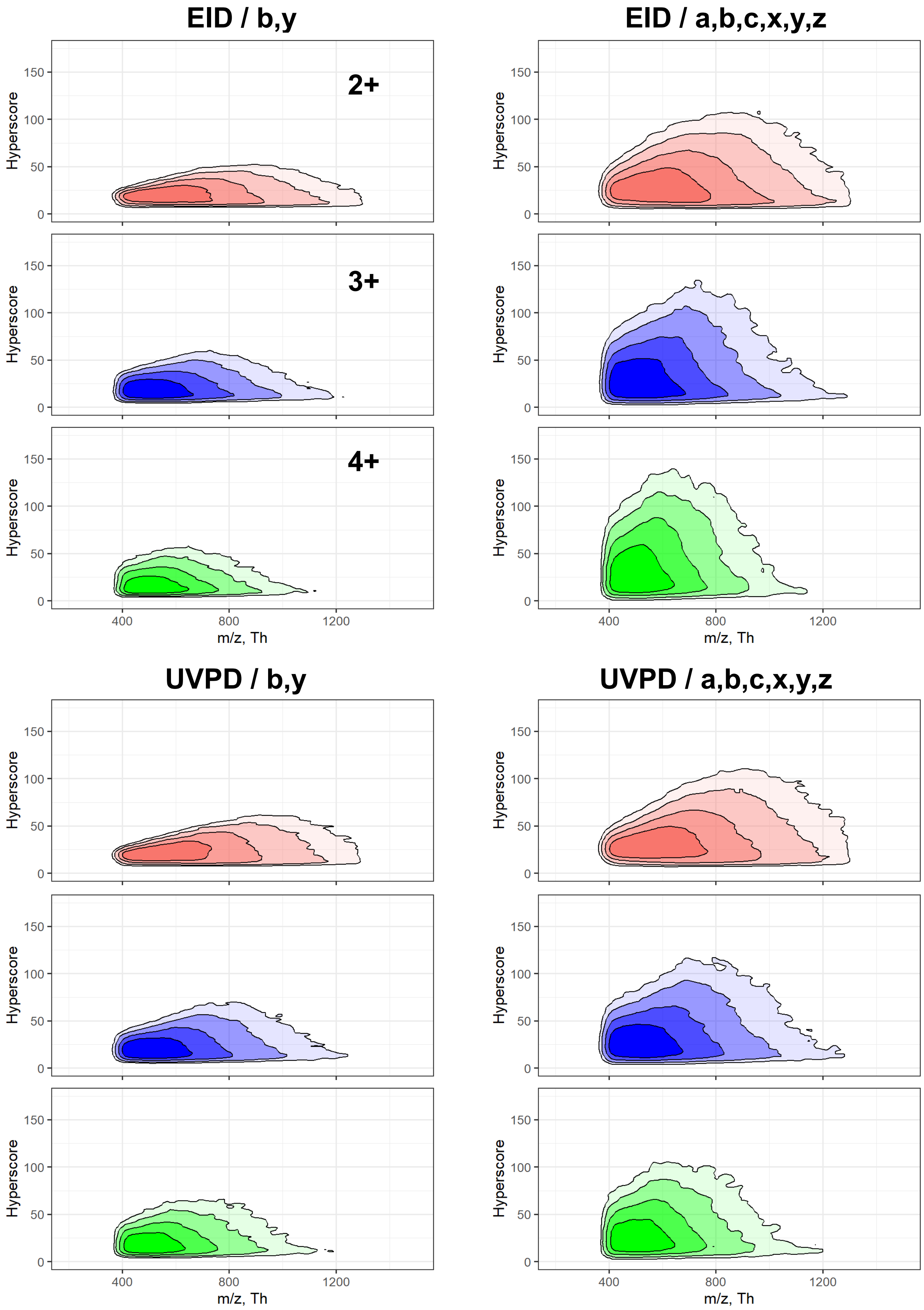

Supplementary Figure S4. Density contour plots of hyperscore distributions against *m/z* values per charge state (by *vs* abcxyz). Density contour plots of hyperscore distributions of 2+ (red), 3+ (blue), and 4+ (green) charge states of unique PSMs (unique combination of amino-acid sequence, charge, and modification selected by highest hyperscore) plotted against *m/z* values of precursors acquired in EID and UVPD using *b* and *y* types of fragments (left) and in EID and UVPD using *a,b,c,x,y,z* types of fragments (right). Contour lines demarcate the smallest regions to contain 50, 80, 95 and 99% of data points.

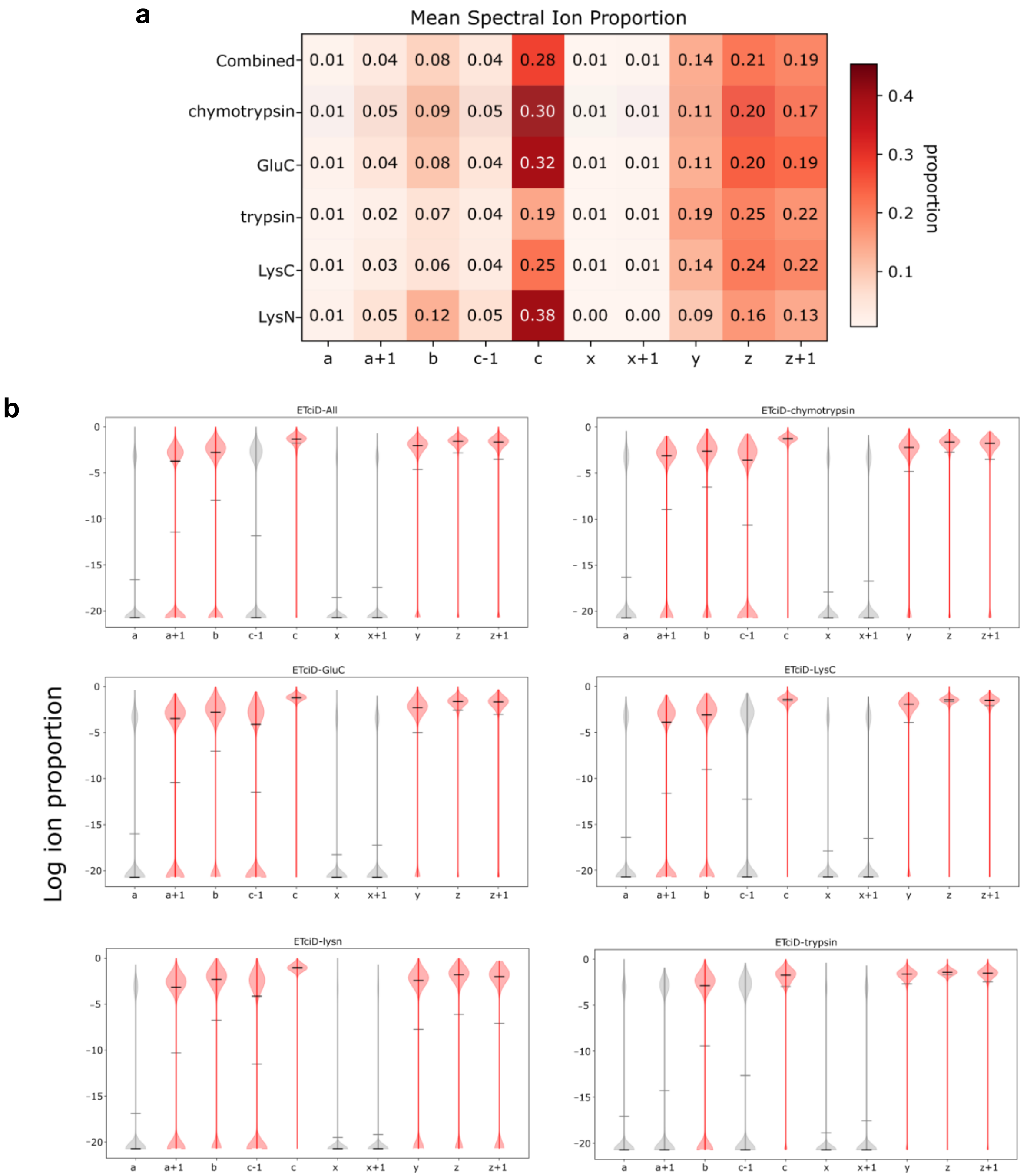

Supplementary Figure S5. Frequencies and log ion proportions in ETciD data. a, Heatmap of average proportions of fragment ion peaks of different types among all annotated peaks in ETciD spectra, not reflecting relative ion intensities. Annotation was performed for 10 ion types: *a*, *a+1*, *b*, *c-1*, *c*, *x*, *x+1*, *y*, *z*, *z+1*. **b,** Log ion proportions of ions annotated in ETciD data. Ions annotated with negligible frequencies (<4% of all annotated ions) are greyed out. Horizontal grey and black lines correspond to the median and mean values, respectively.

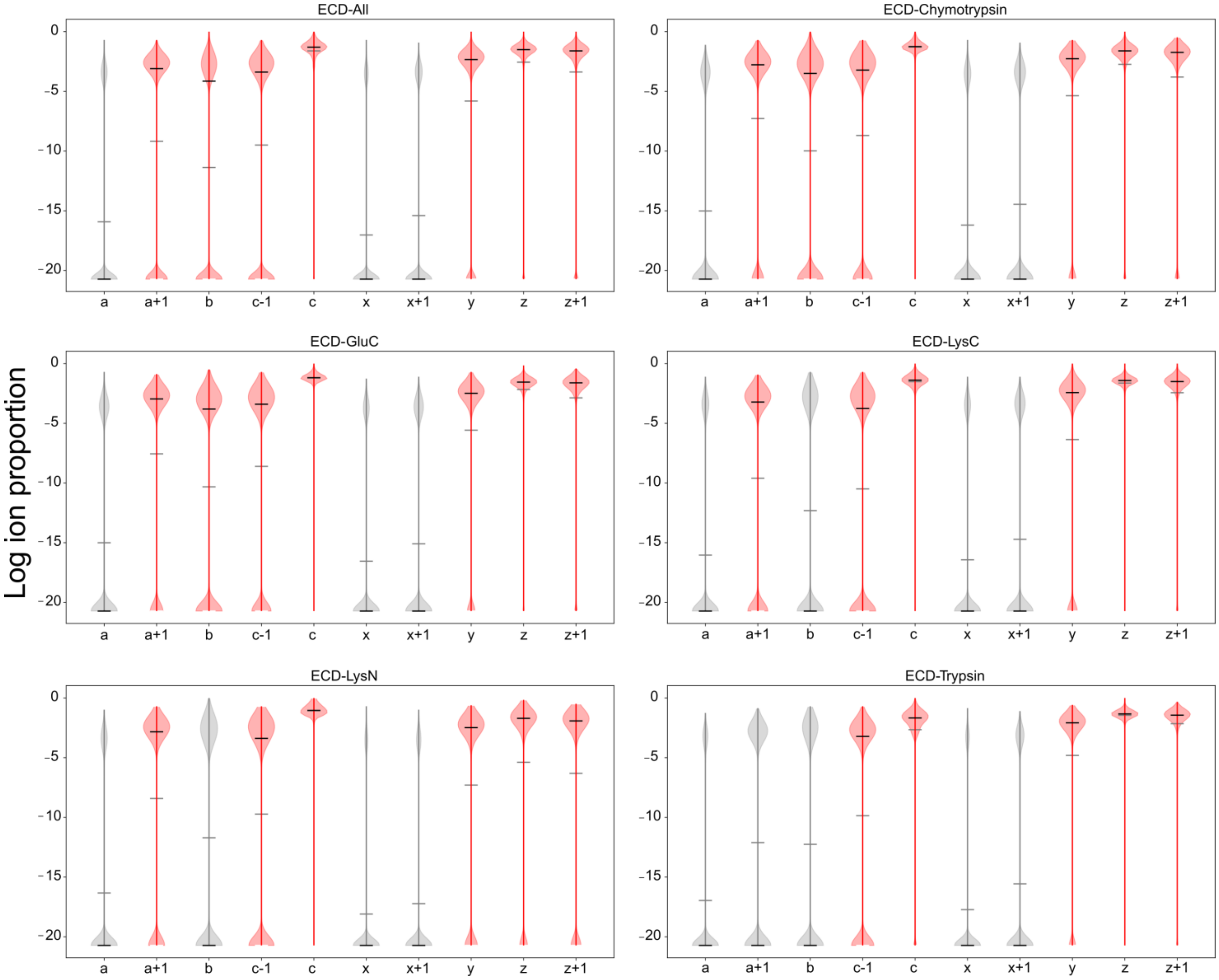

Supplementary Figure S6. Log ion proportions of ions annotated in ECD data. Ions annotated with negligible frequencies (<4% of all annotated ions) are greyed out. Horizontal grey and black lines correspond to the median and mean values, respectively.

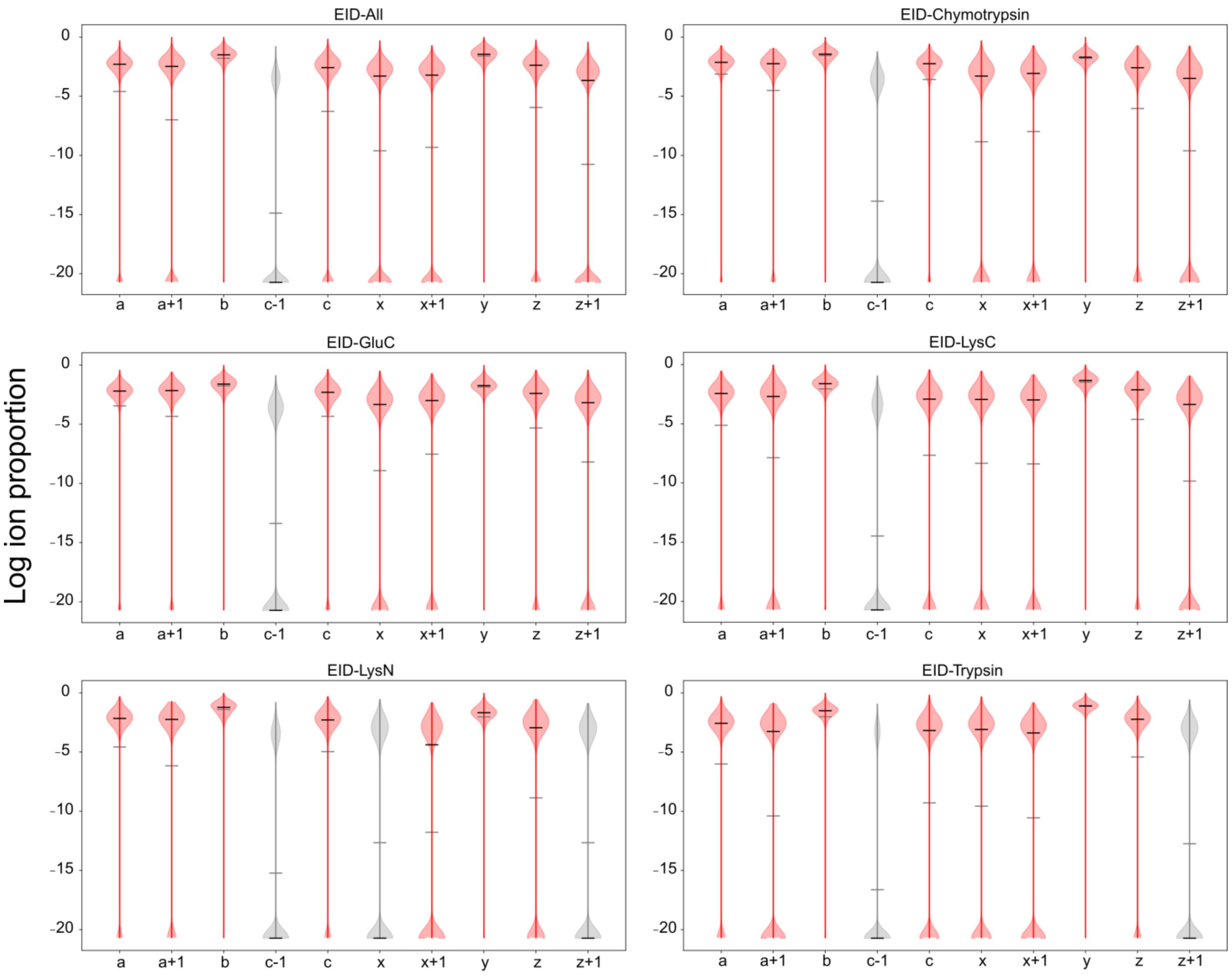

Supplementary Figure S7. Log ion proportions of ions annotated in EID data. Ions annotated with negligible frequencies (<4% of all annotated ions) are greyed out. Horizontal grey and black lines correspond to the median and mean values, respectively.

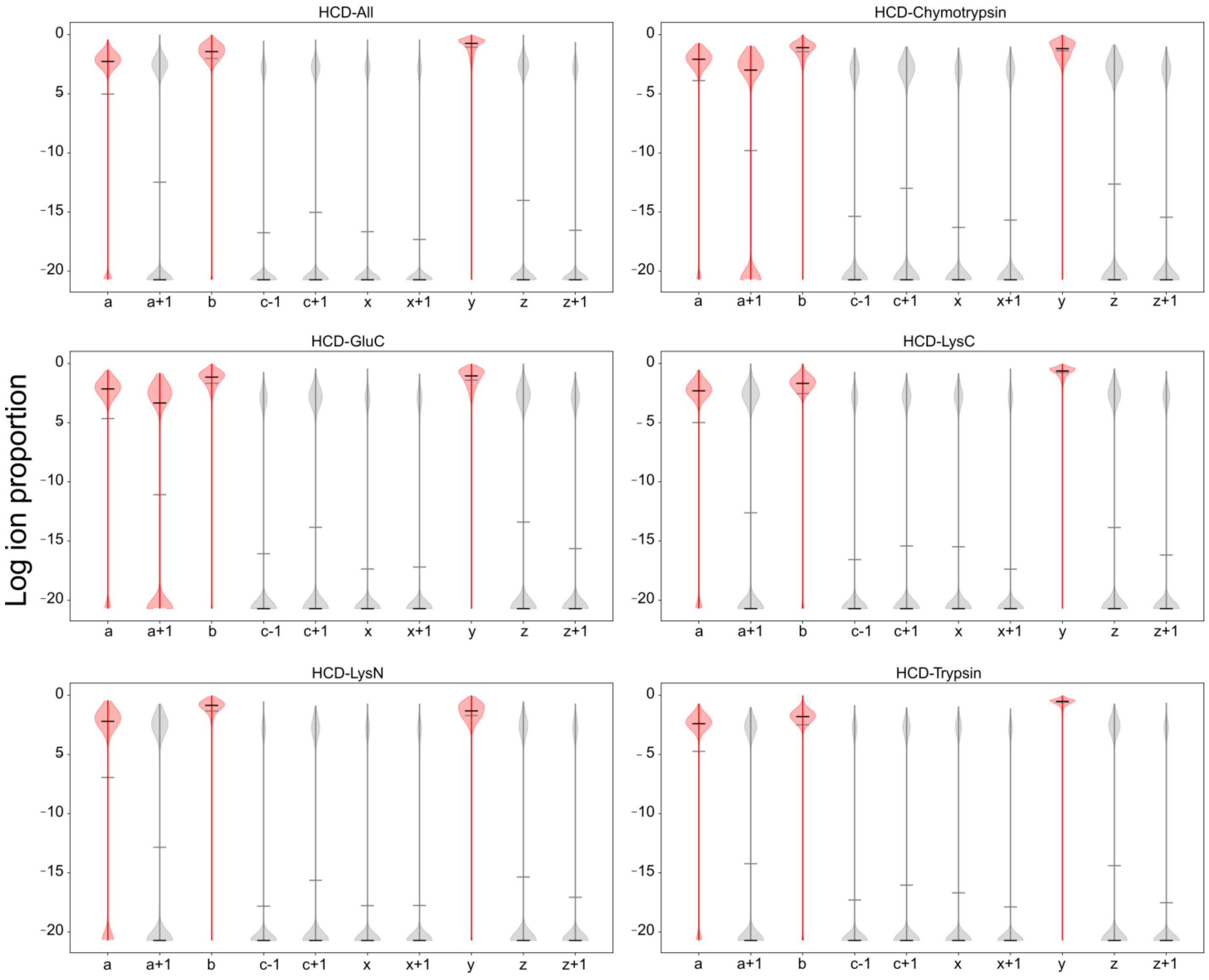

Supplementary Figure S8. Log ion proportions of ions annotated in HCD data. Ions annotated with negligible frequencies (<4% of all annotated ions) are greyed out. Horizontal grey and black lines correspond to the median and mean values, respectively.

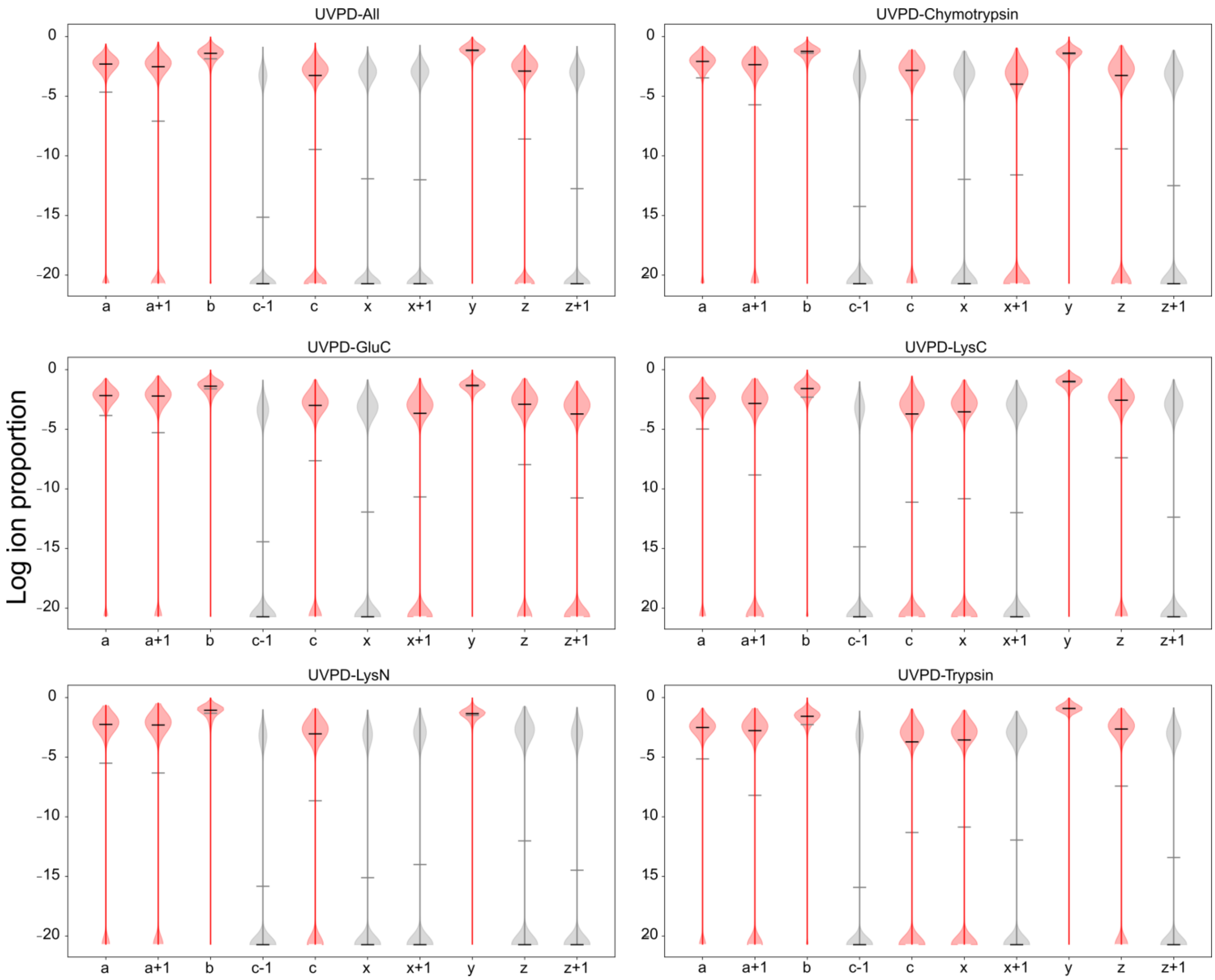

Supplementary Figure S9. Log ion proportions of ions annotated in UVPD data. Ions annotated with negligible frequencies (<4% of all annotated ions) are greyed out. Horizontal grey and black lines correspond to the median and mean values, respectively.

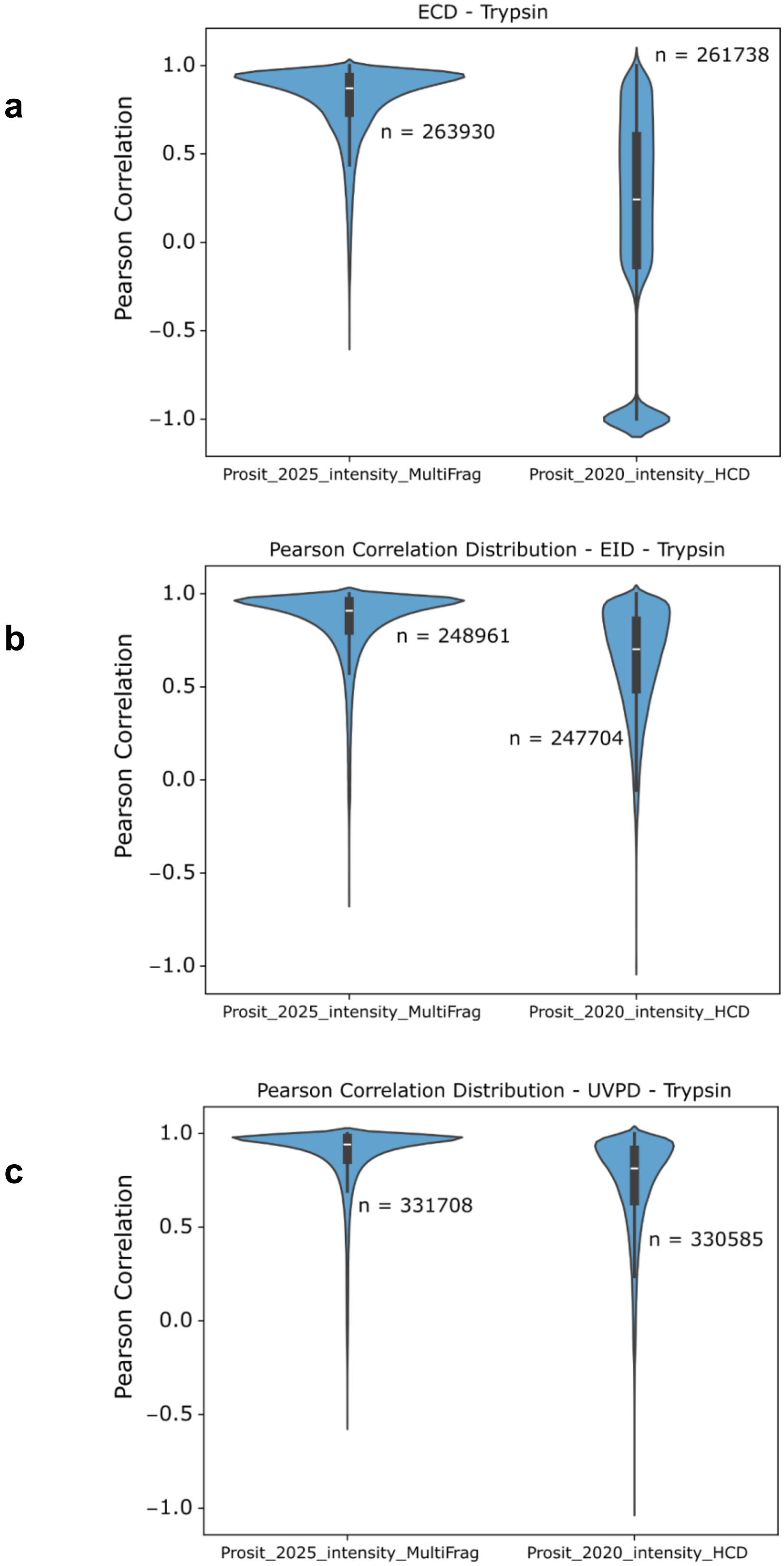

Supplementary Figure S10. Benchmarking the ECD, EID and UVPD datasets against the Multifrag and HCD 2020 Prosit models. Pearson correlation between experimental spectra and spectra predicted by the "Multifrag" (left) and "HCD_2020" [Ref. 73] (right) Prosit models in ECD (**a**), EID (**b**) and UVPD (**c**) datasets. The distributions contain box and whisker bars. Distributions protruding beyond 1.0 or -1.0 are plotting artefacts.

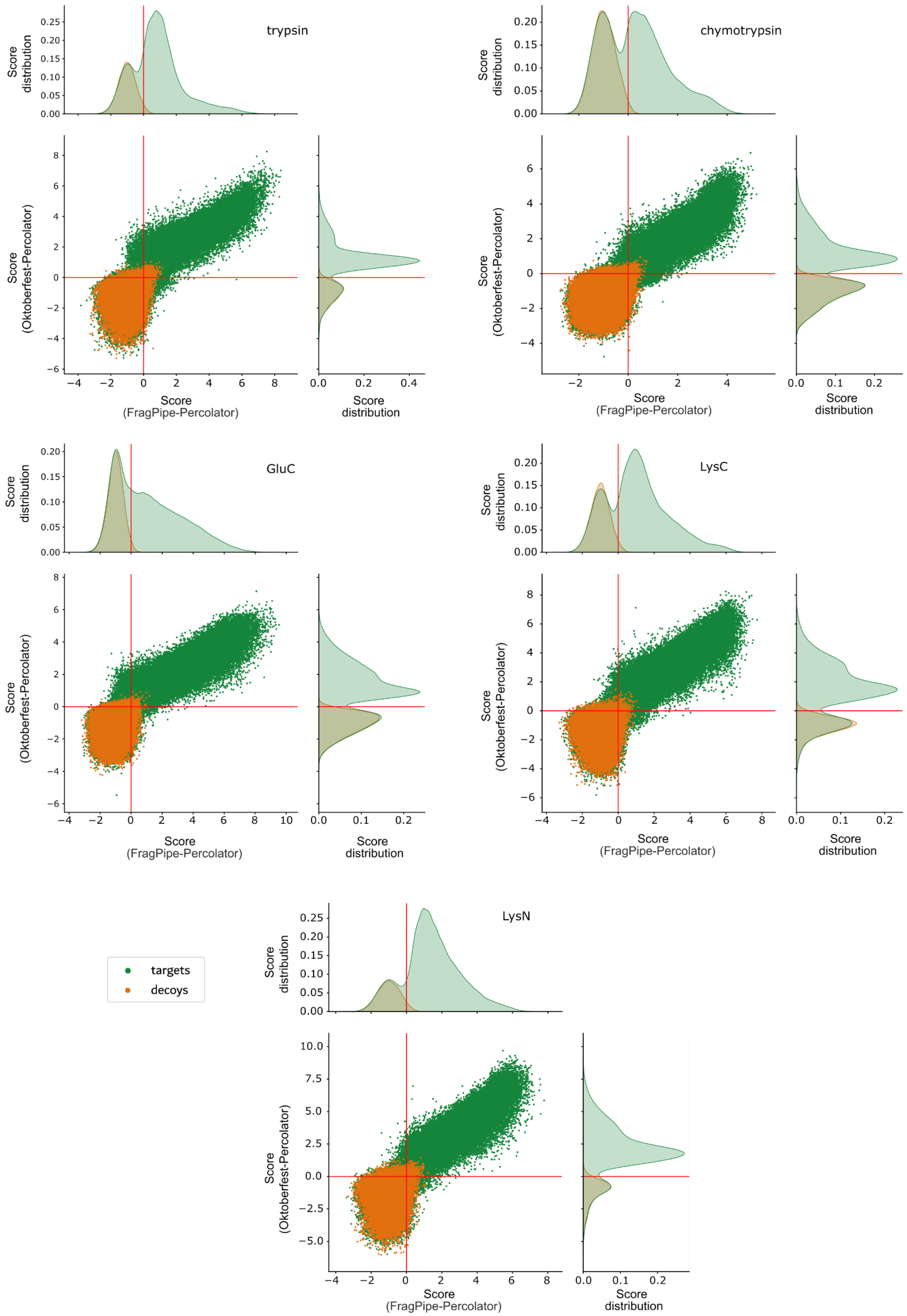

Supplementary Figure S11. Oktoberfest rescoring separates target from decoy (ECD). Correlation of Percolator scores for all target (green) and decoy (orange) PSMs in ECD data acquired from rescoring the MSFragger (top) and Oktoberfest (right) sets of scores plotted per enzyme. The red lines indicate the 1% PSM-level FDR cutoffs in MSFragger and Oktoberfest score distributions.

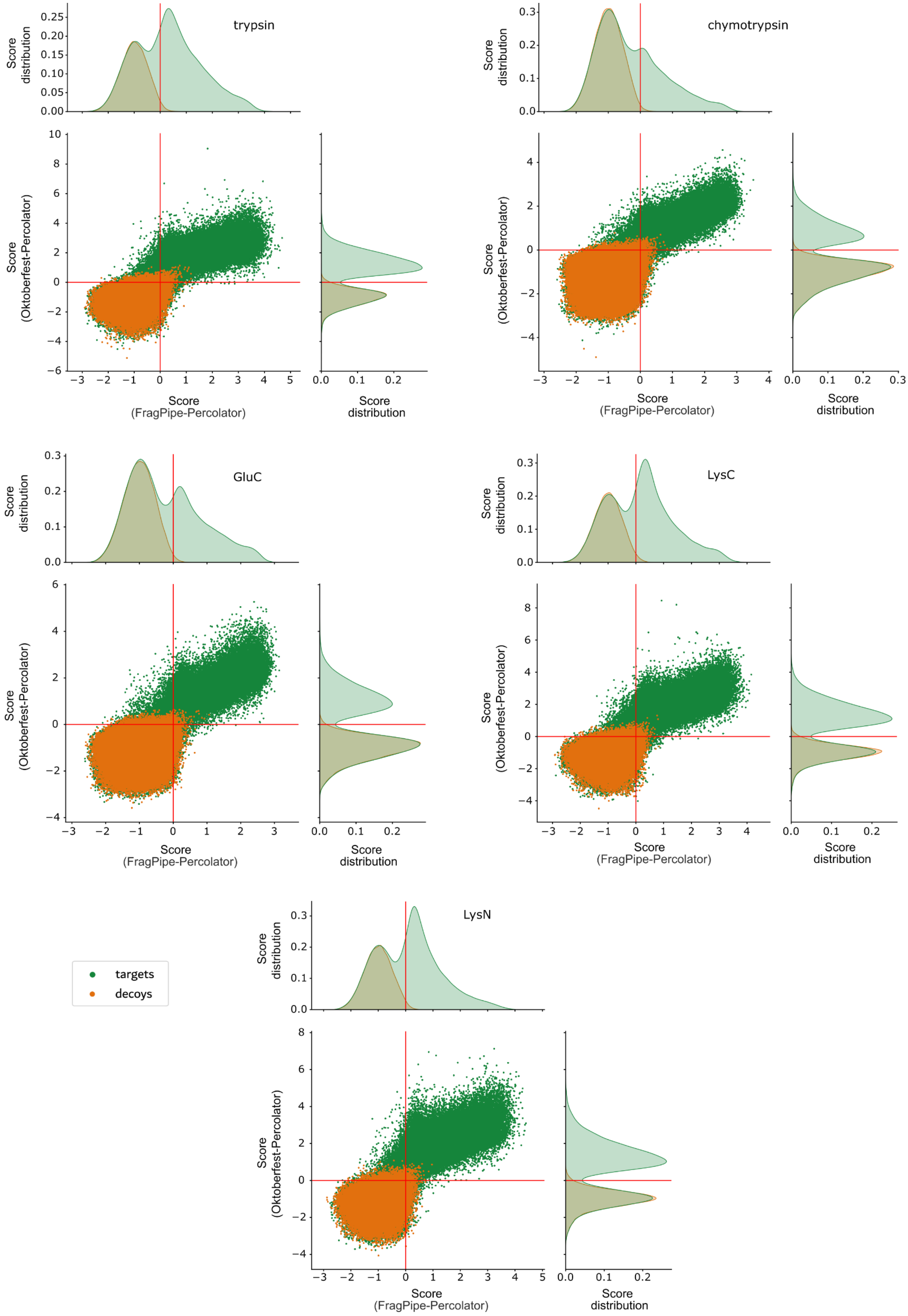

Supplementary Figure S12. Oktoberfest rescoring separates target from decoy (EID). Correlation of Percolator scores for all target (green) and decoy (orange) PSMs in EID data acquired from rescoring the MSFragger (top) and Oktoberfest (right) sets of scores plotted per enzyme. The red lines indicate the 1% PSM-level FDR cutoffs in MSFragger and Oktoberfest score distributions.

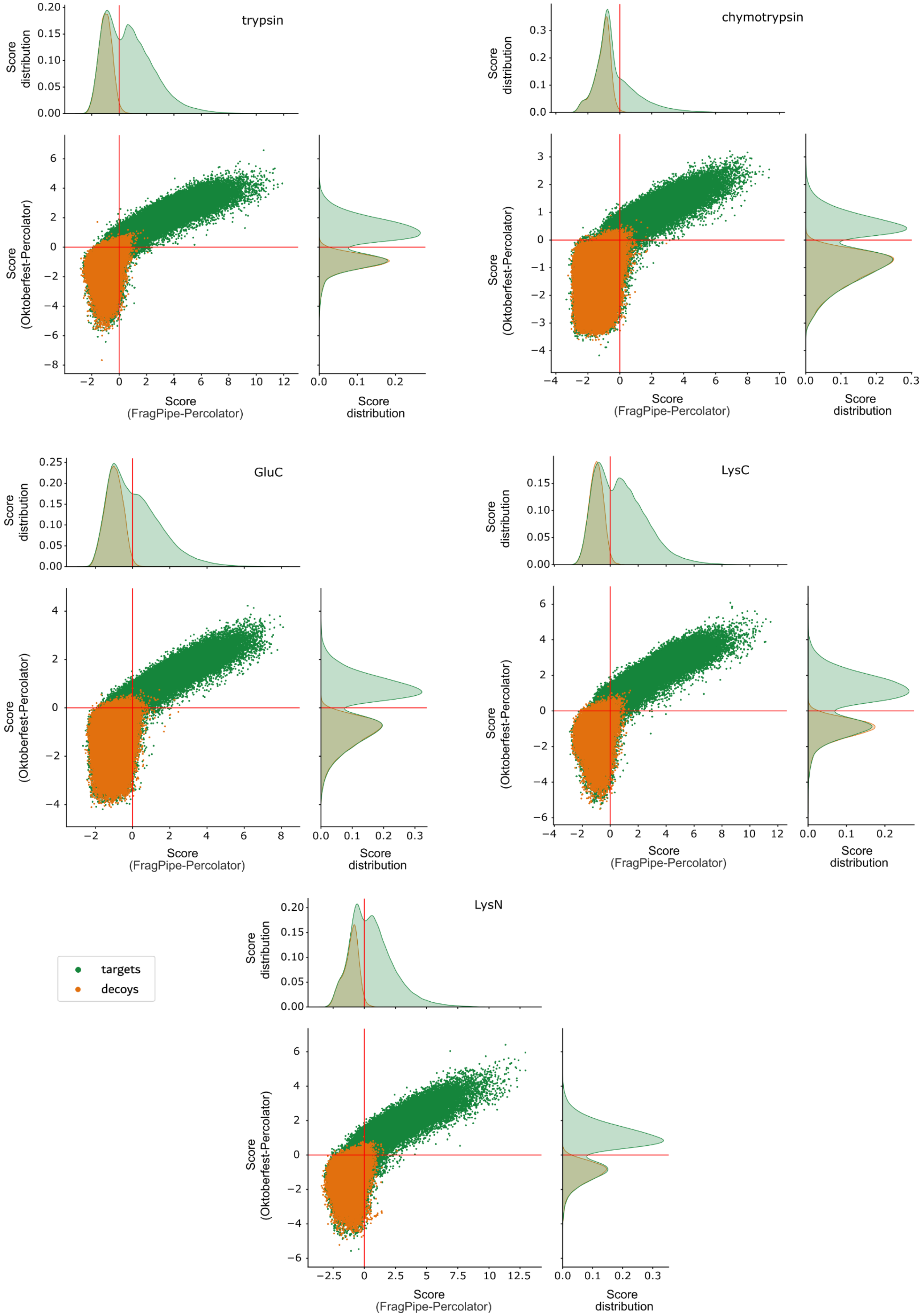

Supplementary Figure S13. Oktoberfest rescoring separates target from decoy (HCD). Correlation of Percolator scores for all target (green) and decoy (orange) PSMs in HCD data acquired from rescoring the MSFragger (top) and Oktoberfest (right) sets of scores plotted per enzyme. The red lines indicate the 1% PSM-level FDR cutoffs in MSFragger and Oktoberfest score distributions.

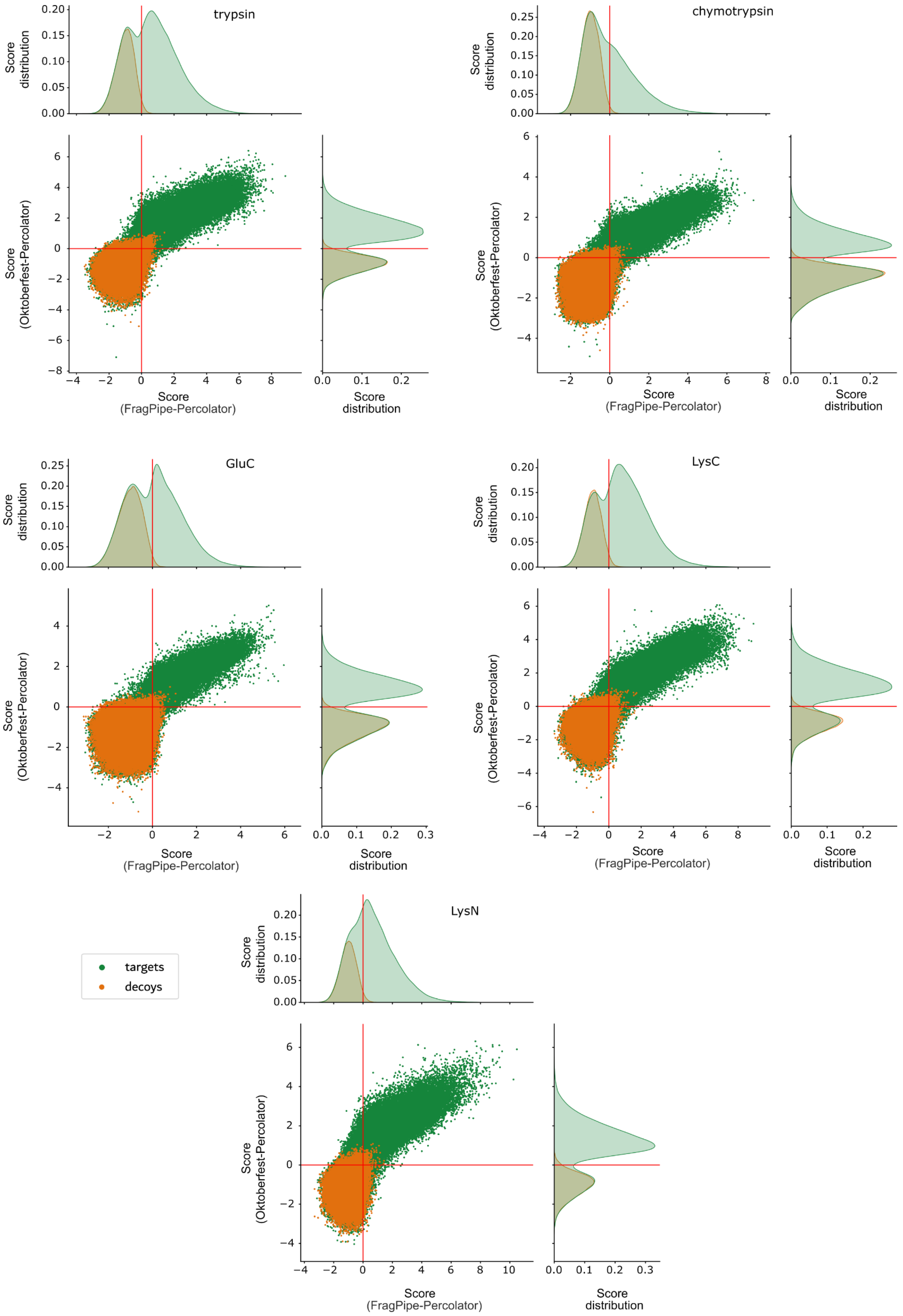

Supplementary Figure S14. Oktoberfest rescoring separates target from decoy (UVPD). Correlation of Percolator scores for all target (green) and decoy (orange) PSMs in UVPD data acquired from rescoring the MSFragger (top) and Oktoberfest (right) sets of scores plotted per enzyme. The red lines indicate the 1% PSM-level FDR cutoffs in MSFragger and Oktoberfest score distributions.

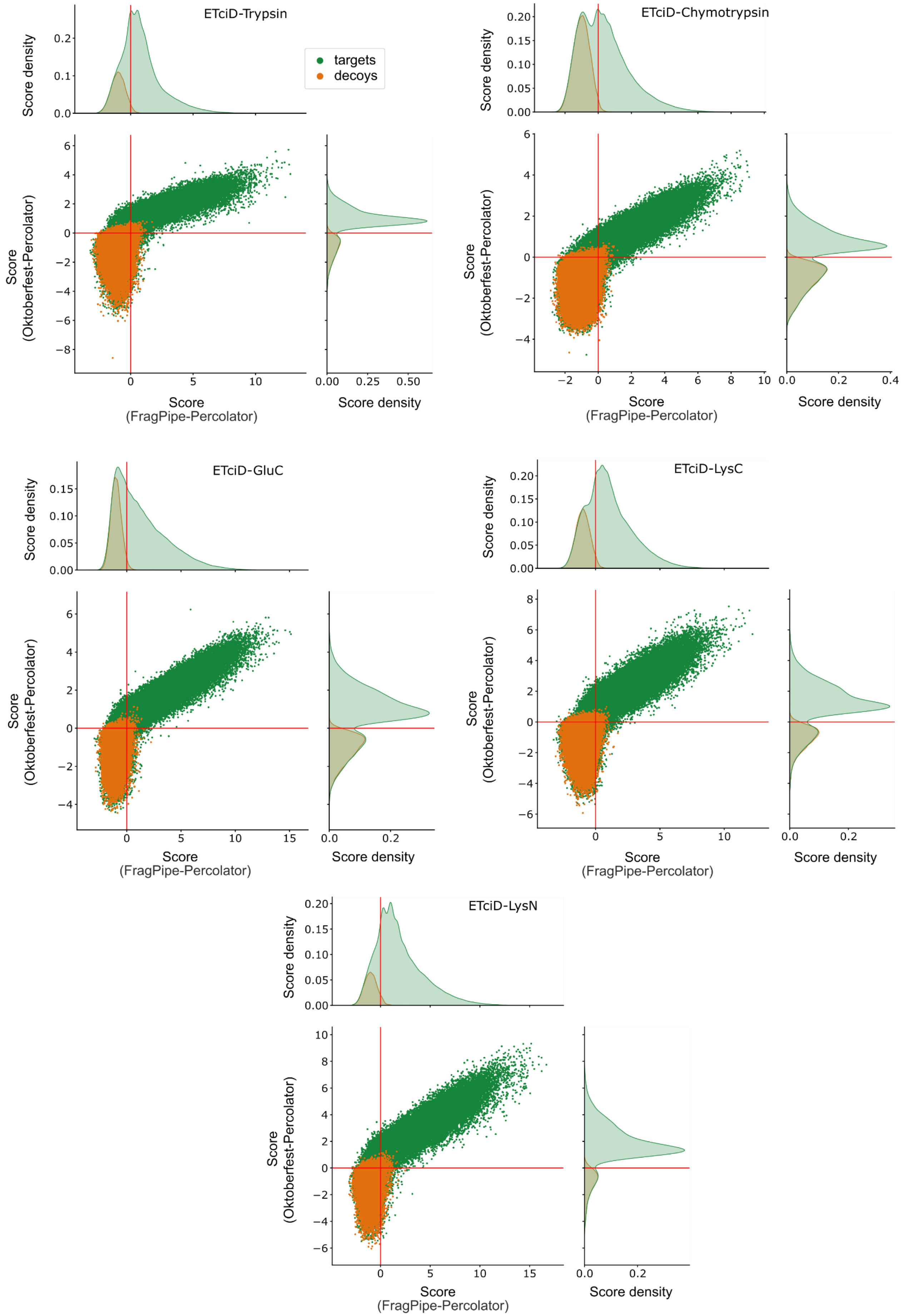

Supplementary Figure S15. Oktoberfest rescoring separates target from decoy (ETciD). Correlation of Percolator scores for all target (green) and decoy (orange) PSMs in ETciD data acquired from rescoring the MSFragger (top) and Oktoberfest (right) sets of scores plotted per enzyme. The red lines indicate the 1% PSM-level FDR cutoffs in MSFragger and Oktoberfest score distributions.

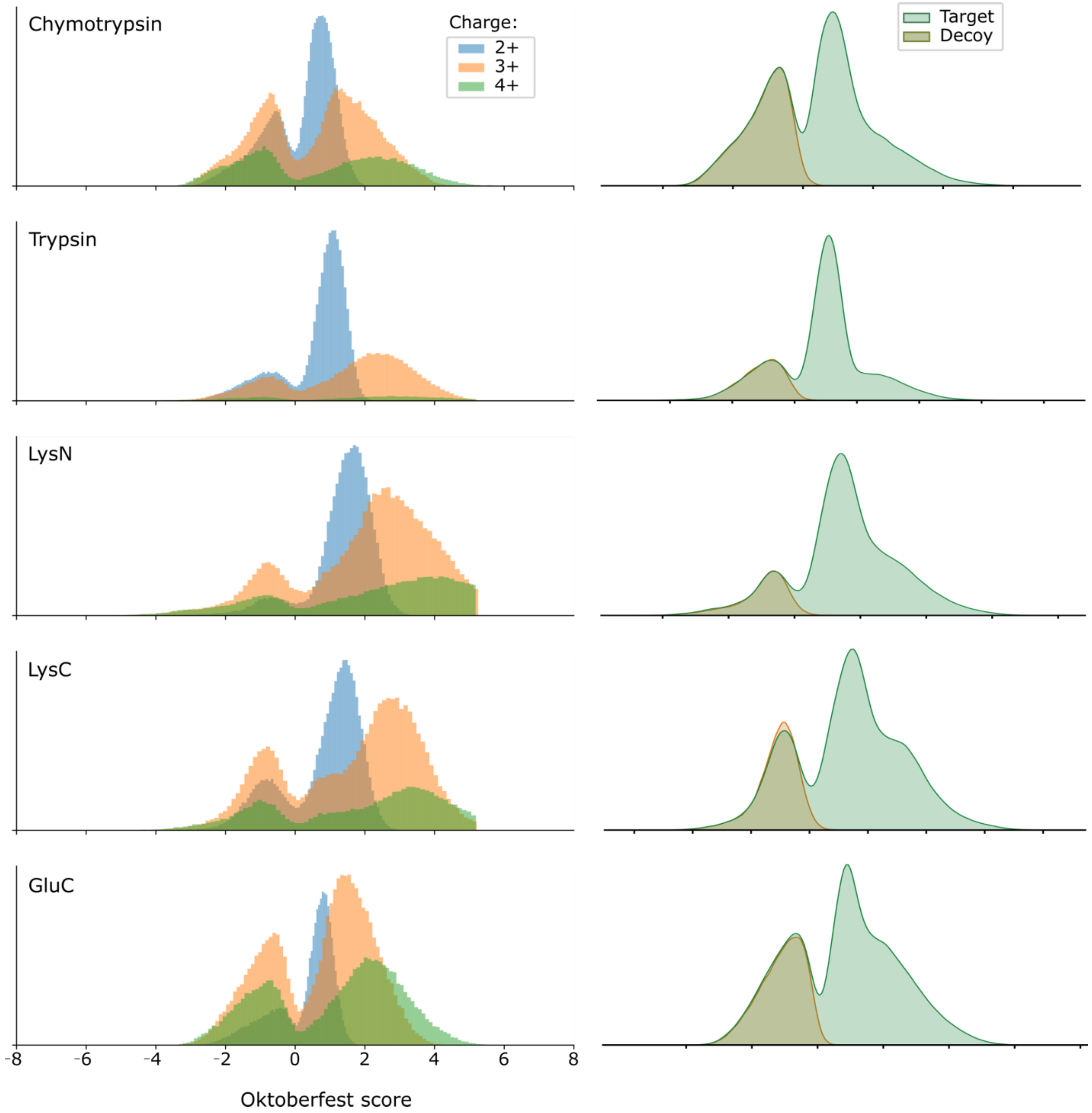

Supplementary Figure S16. Distributions of target PSMs in ECD are charge-separated after rescoring in Oktoberfest. Left: PSM-level distributions of Oktoberfest scores separated by charge state of precursors plotted for ECD data per enzyme. **Right**: the same distributions summed up and split between decoy and target (taken from Supplementary Fig. S11)

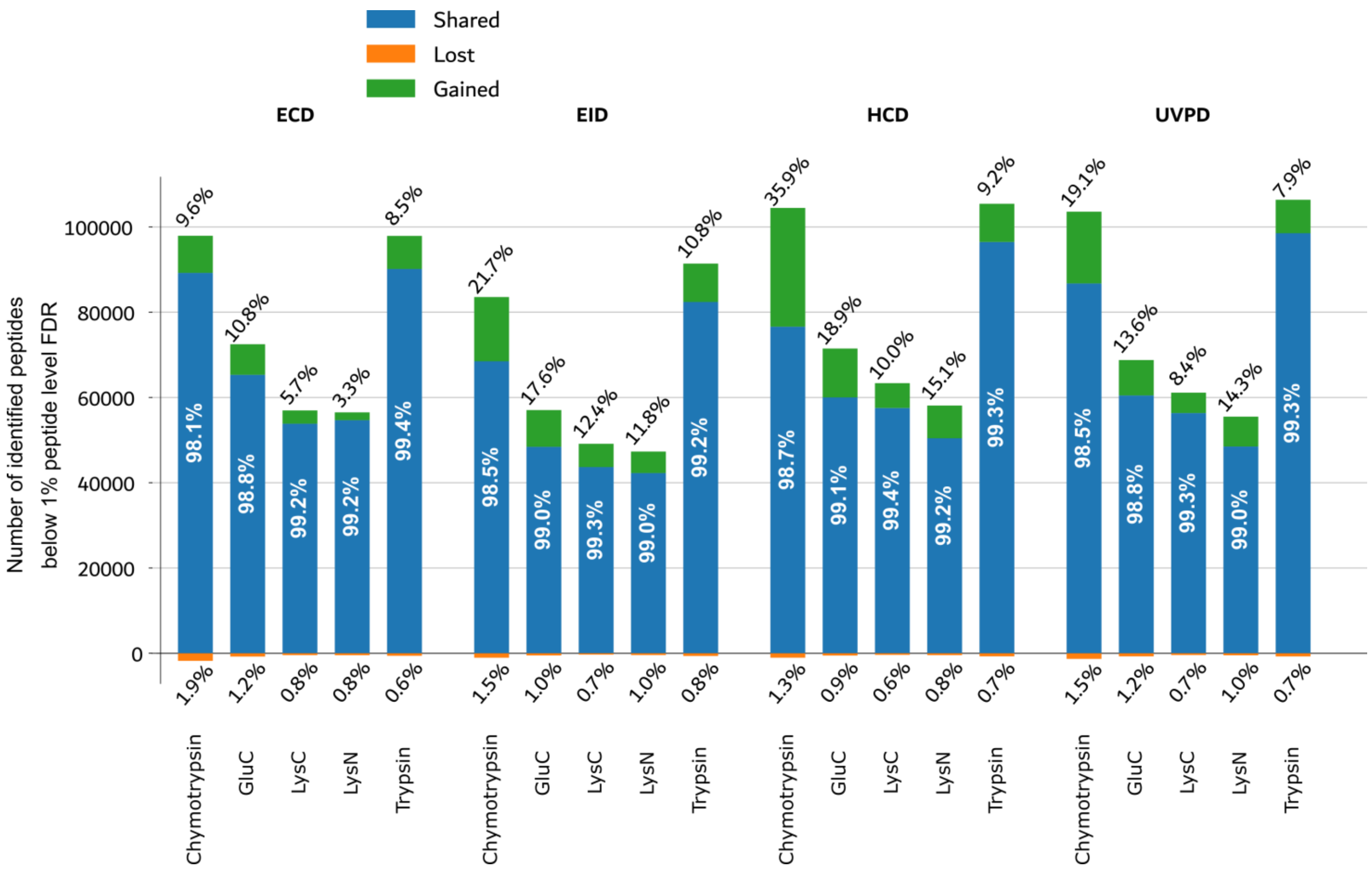

Supplementary Figure S17. Gain/share/loss at the peptide level (Oktoberfest vs MSFragger). Numbers of shared (blue), gained (green), and lost (orange) peptides identified at 1% peptide-level FDR using the Oktoberfest set of scores compared to original MSFragger search of ECD, EID, HCD and UVPD data per enzyme.

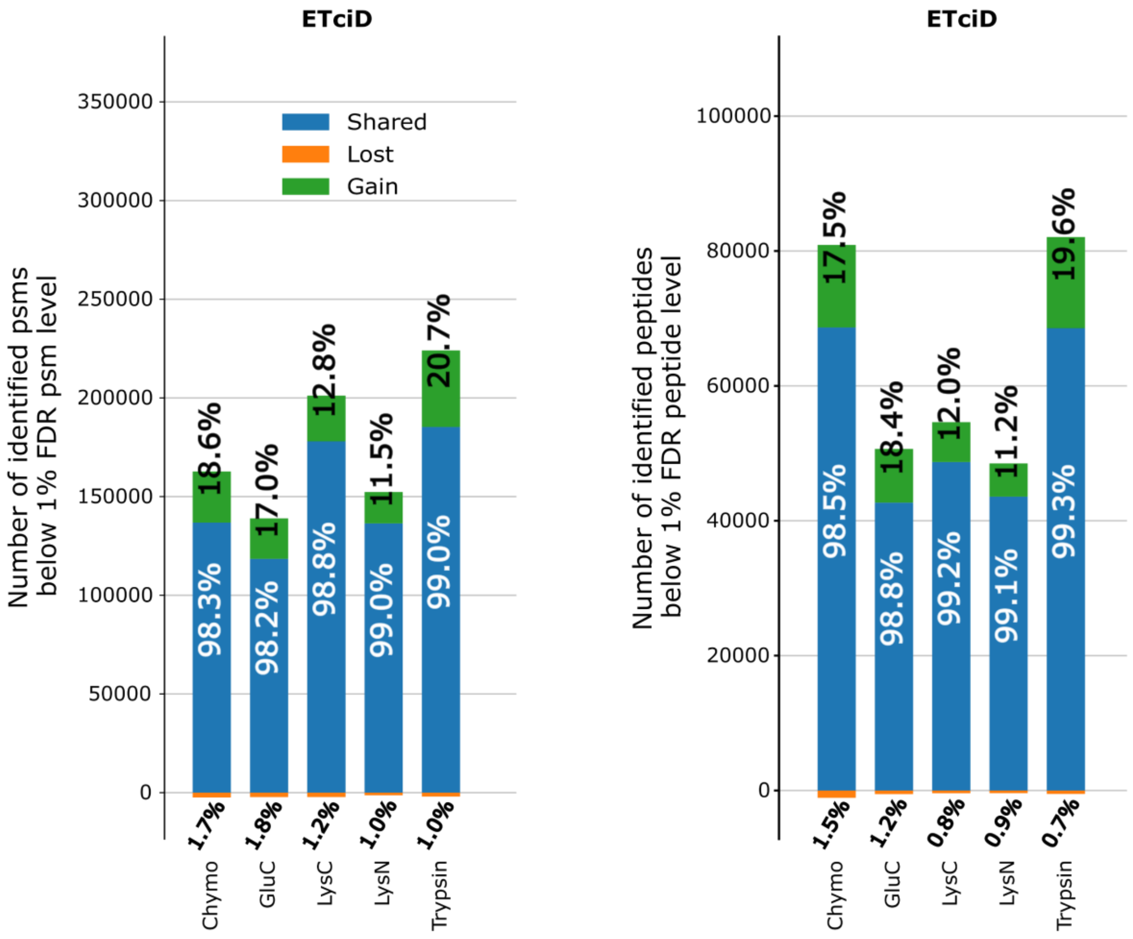

Supplementary Figure S18. Gain/share/loss (Oktoberfest vs MSFragger) in the ETciD data. Numbers of shared (blue), gained (green), and lost (orange) PSMs (left) and peptides (right) identified at 1% FDR using the Oktoberfest set of scores compared to original MSFragger search of ETciD data per enzyme. Chymo: chymotrypsin.

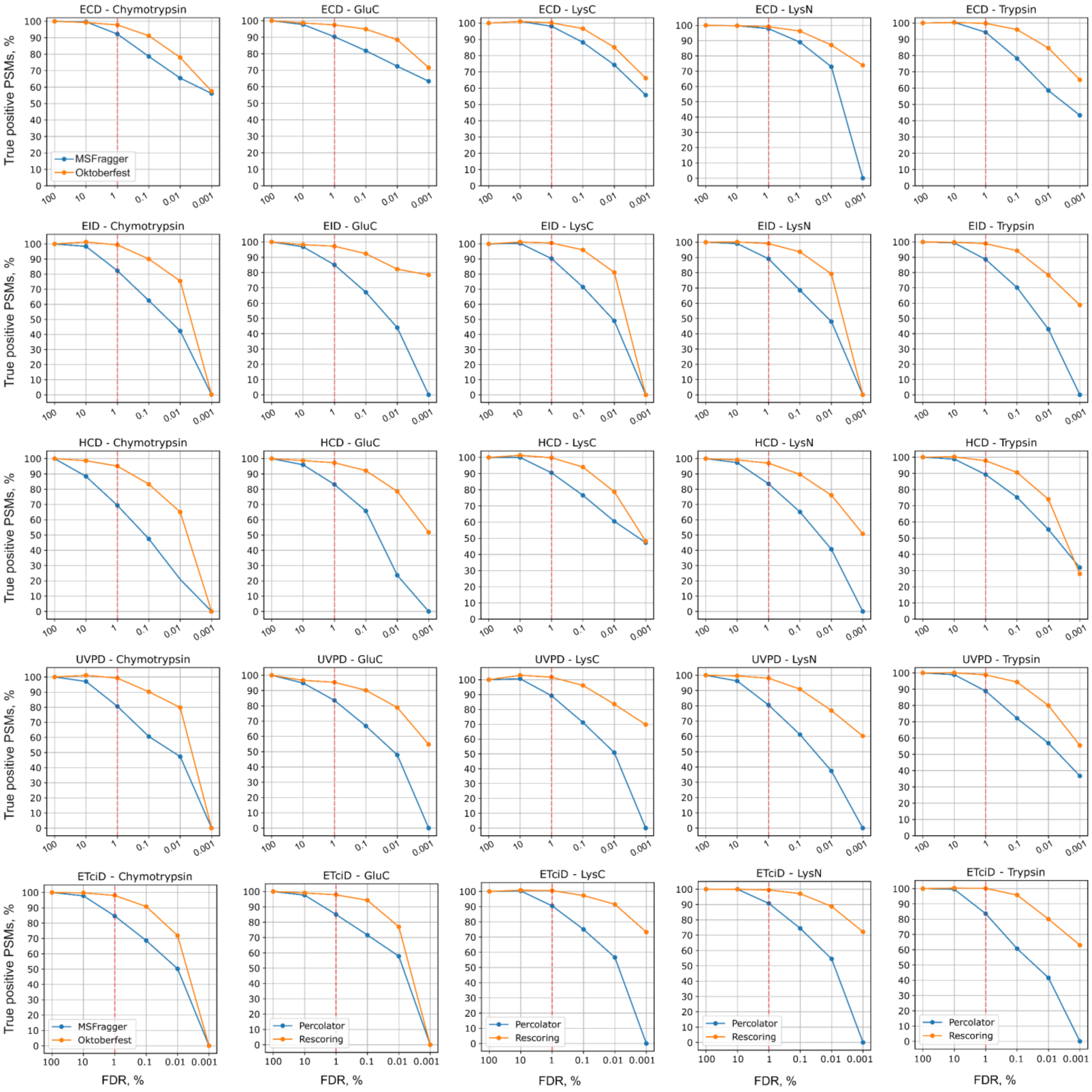

Supplementary Figure S19. Identification of estimated true positives at different FDR thresholds per enzyme. Proportions of the estimated numbers of true positive PSMs to the maximum possible number of true positive PSMs acquired using original MSFragger and Oktoberfest scores at different values of PSM-level FDR for each fragmentation technique, per enzyme.

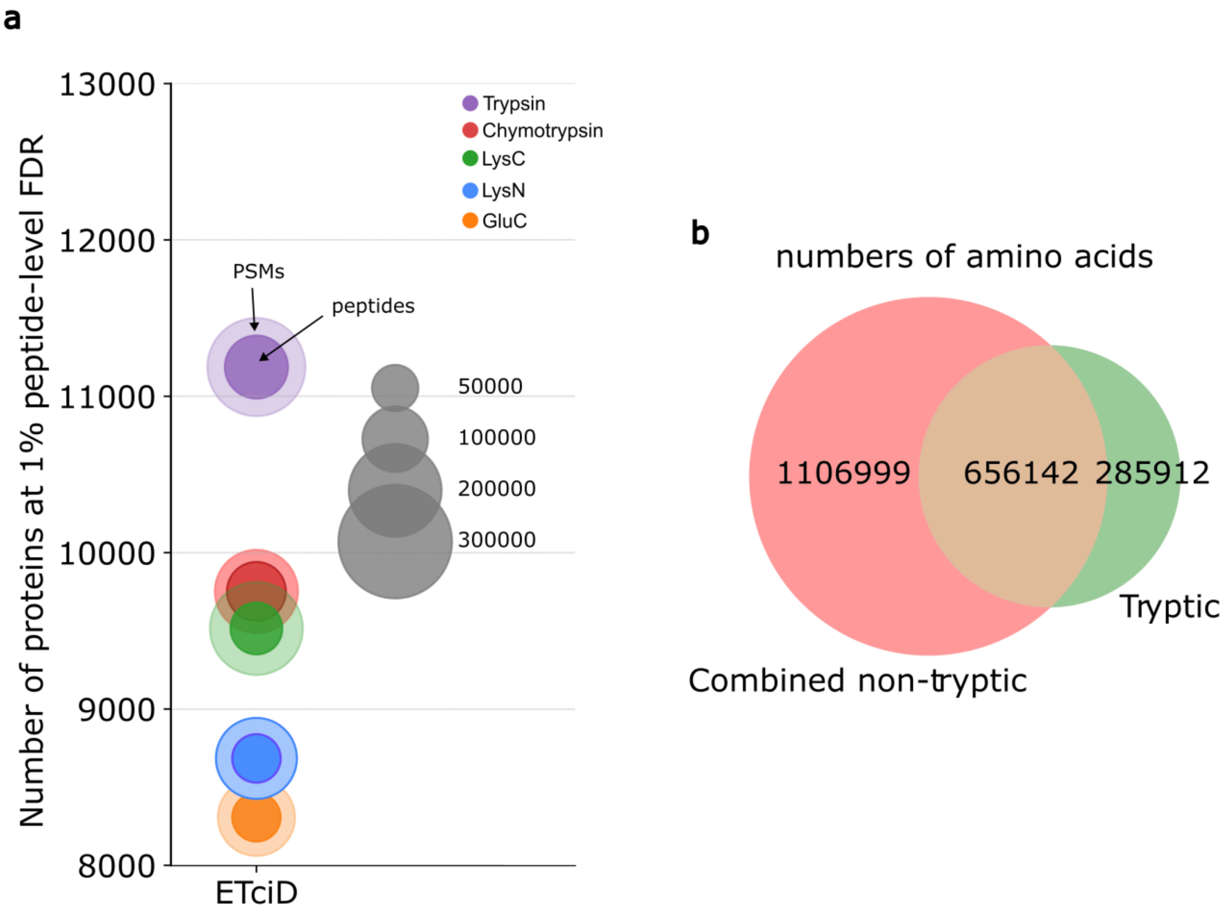

Supplementary Figure S20. Performance of ETciD in DDA searches. a, Numbers of PSMs (outer circles), peptides (inner circles) and proteins (y-axis) identified in ETciD data using different enzymes. **b,** Venn diagram of the total numbers of unique amino acids observed in tryptic datasets (green) and all non-tryptic datasets combined (red) using ETciD.

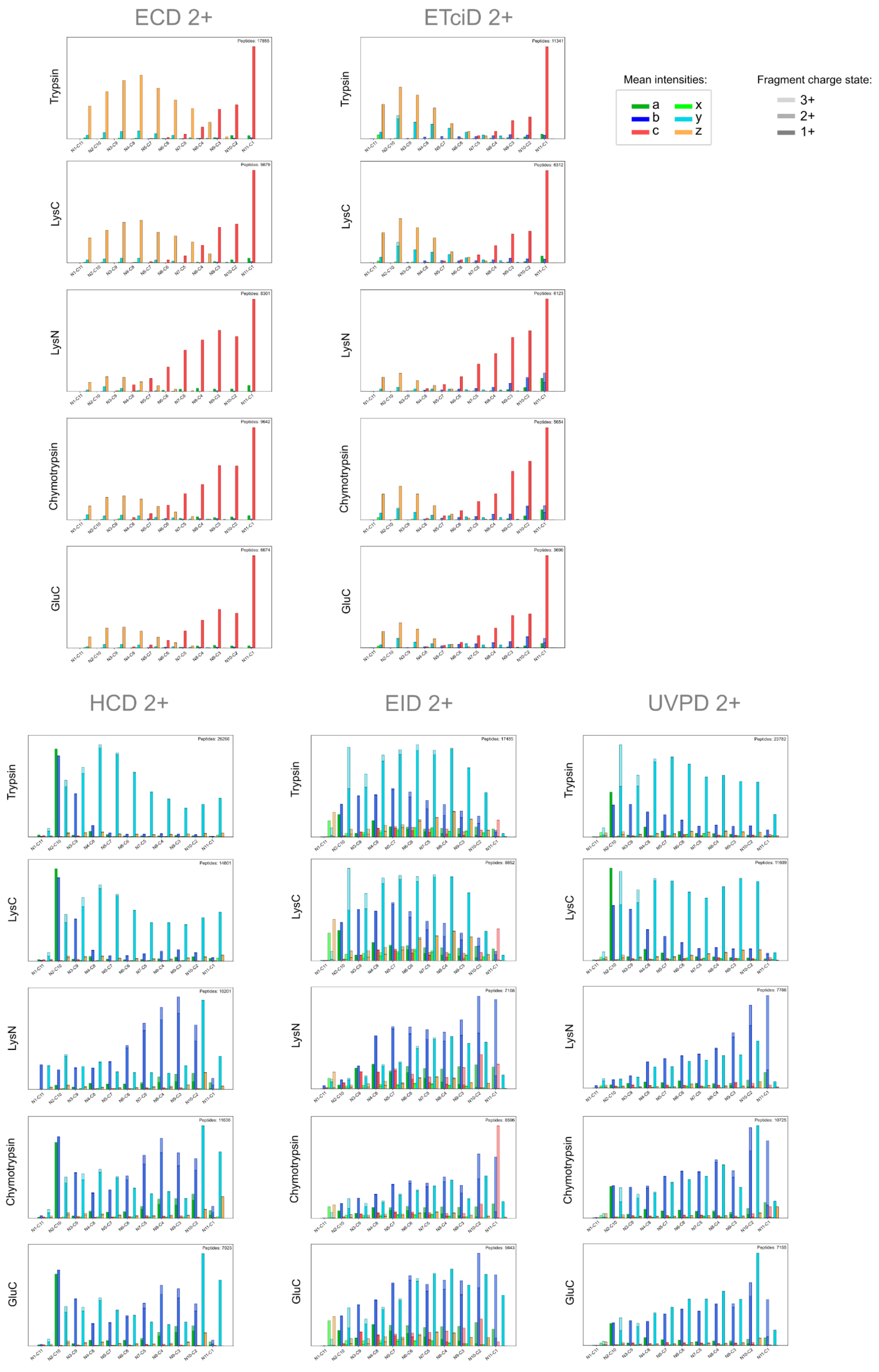

Supplementary Figure S21. Mean normalised intensities of fragments in doubly charged precursors of 12-amino-acid-long peptides. Mean relative intensities of non-zero *a,b,c,x,y,z* ions across all 12-amino-acid-long doubly charged precursors acquired in ECD, ETciD, HCD, EID and UVPD experiments, plotted per enzyme. N# and C# indicate #-th N- and C-terminal fragment, respectively.

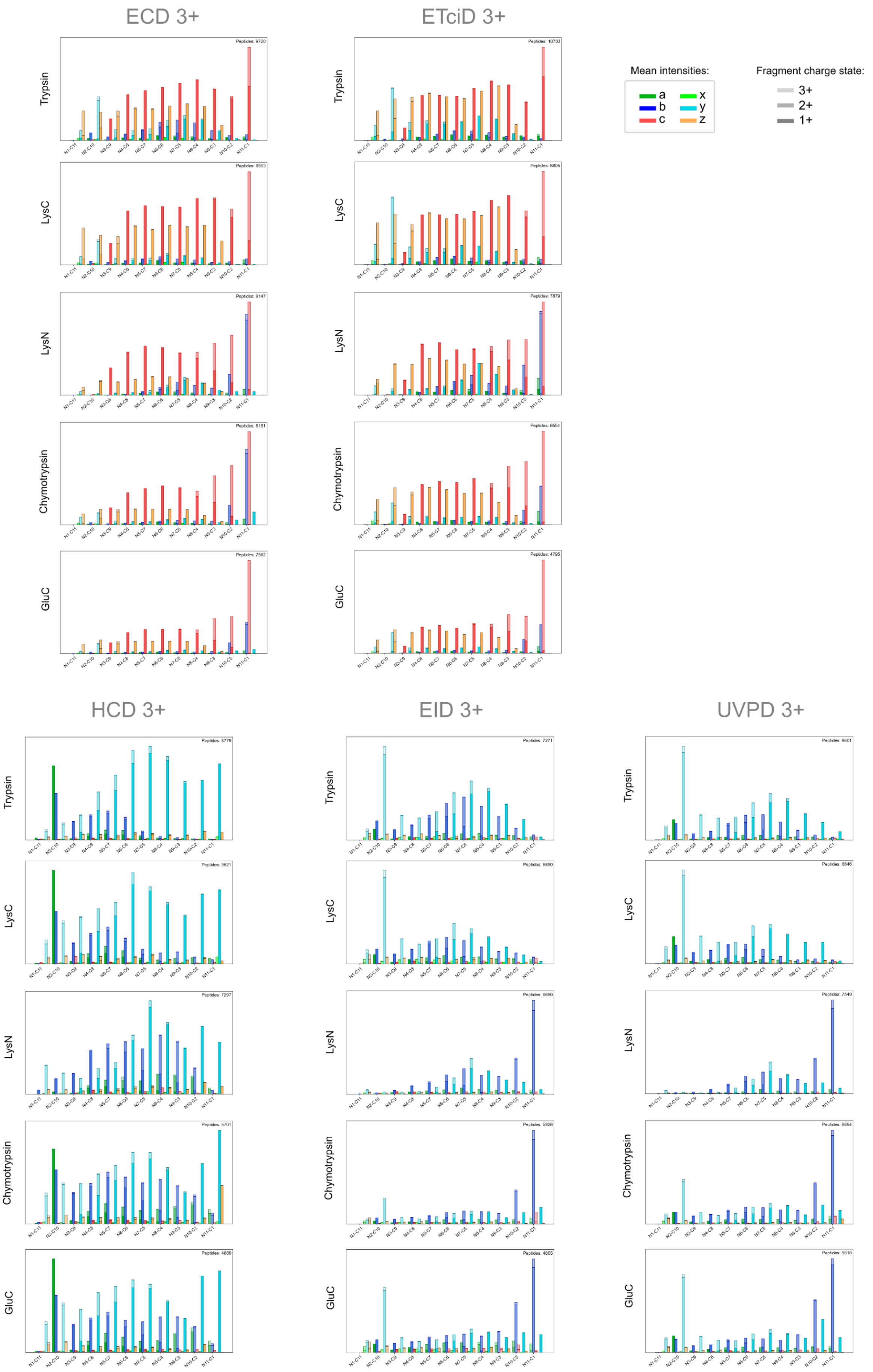

Supplementary Figure S22. Mean normalised intensities of fragments in triply charged precursors of 12-amino-acid-long peptides. Mean relative intensities of non-zero *a,b,c,x,y,z* ions across all 12-amino-acid-long triply charged precursors acquired in ECD, ETciD, HCD, EID and UVPD experiments, plotted per enzyme. N# and C# indicate #-th N- and C-terminal fragment, respectively.

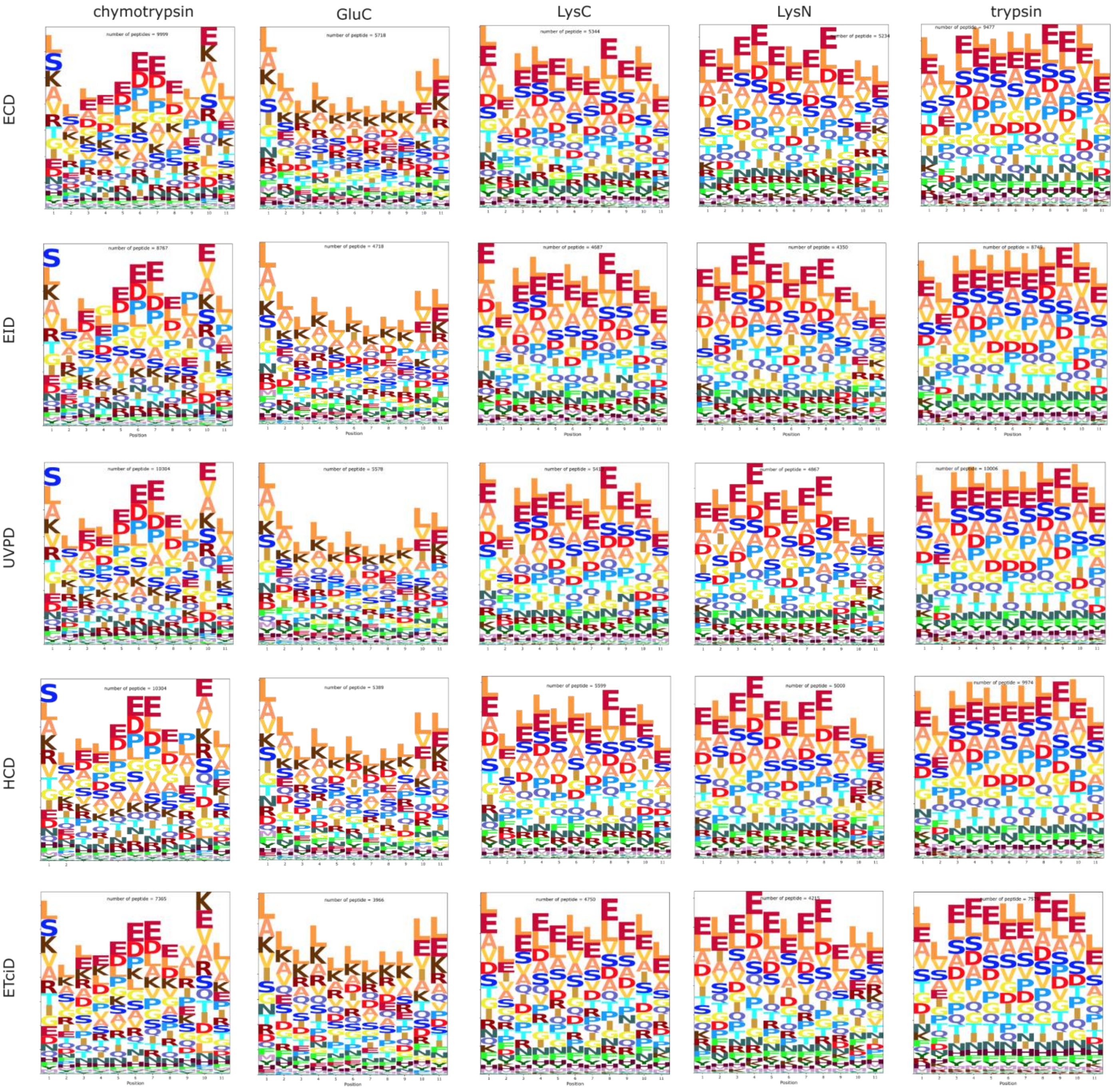

Supplementary Figure S23. Sequence logos of all 12-amino-acid-long peptides. Generated per fragmentation technique per enzyme for all identified 12-amino-acid-long peptides.

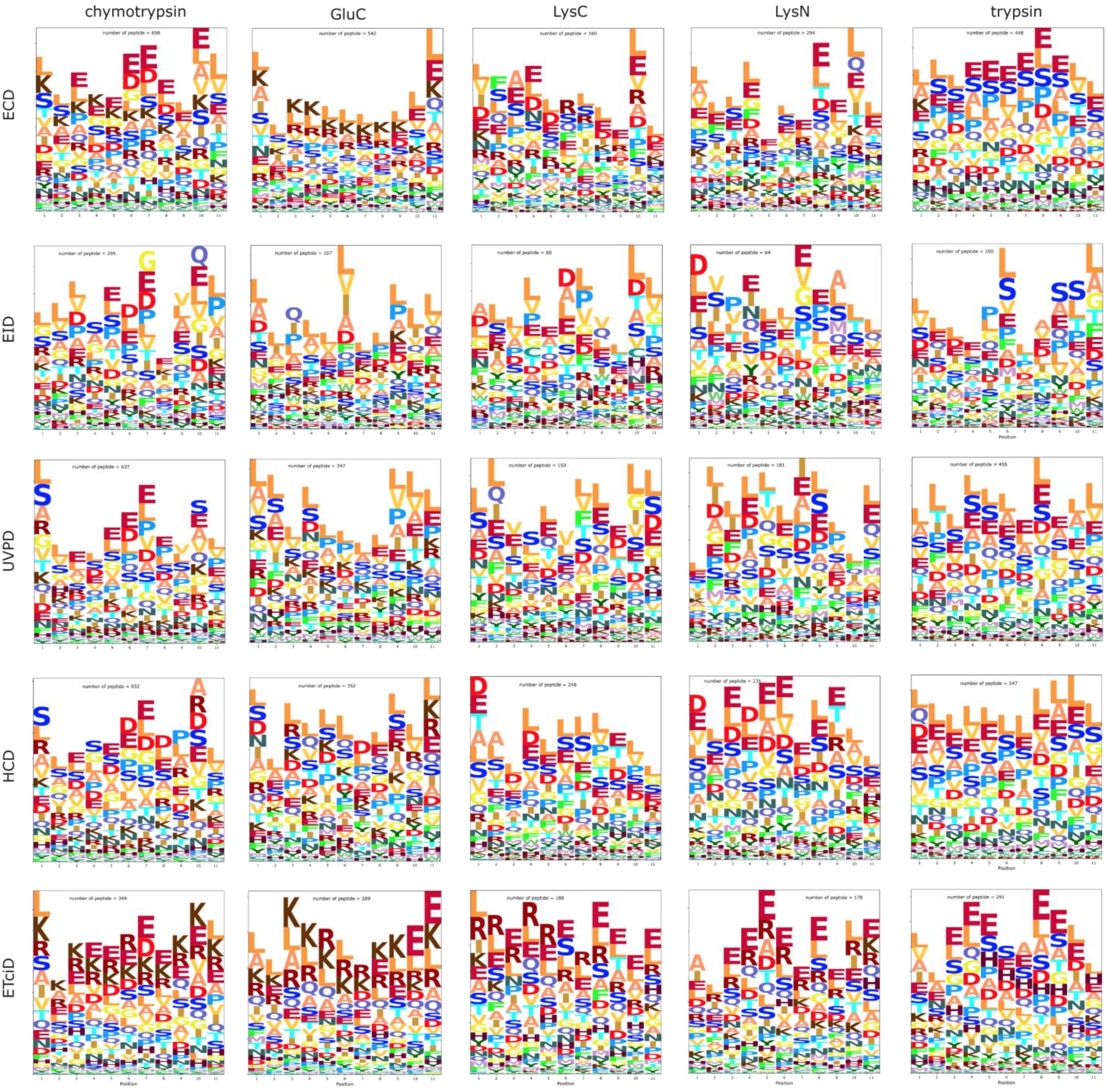

Supplementary Figure S24. Sequence logos of unique 12-amino-acid-long peptides. Generated per fragmentation technique per enzyme for 12-amino-acid-long peptides unique to the fragmentation technique.

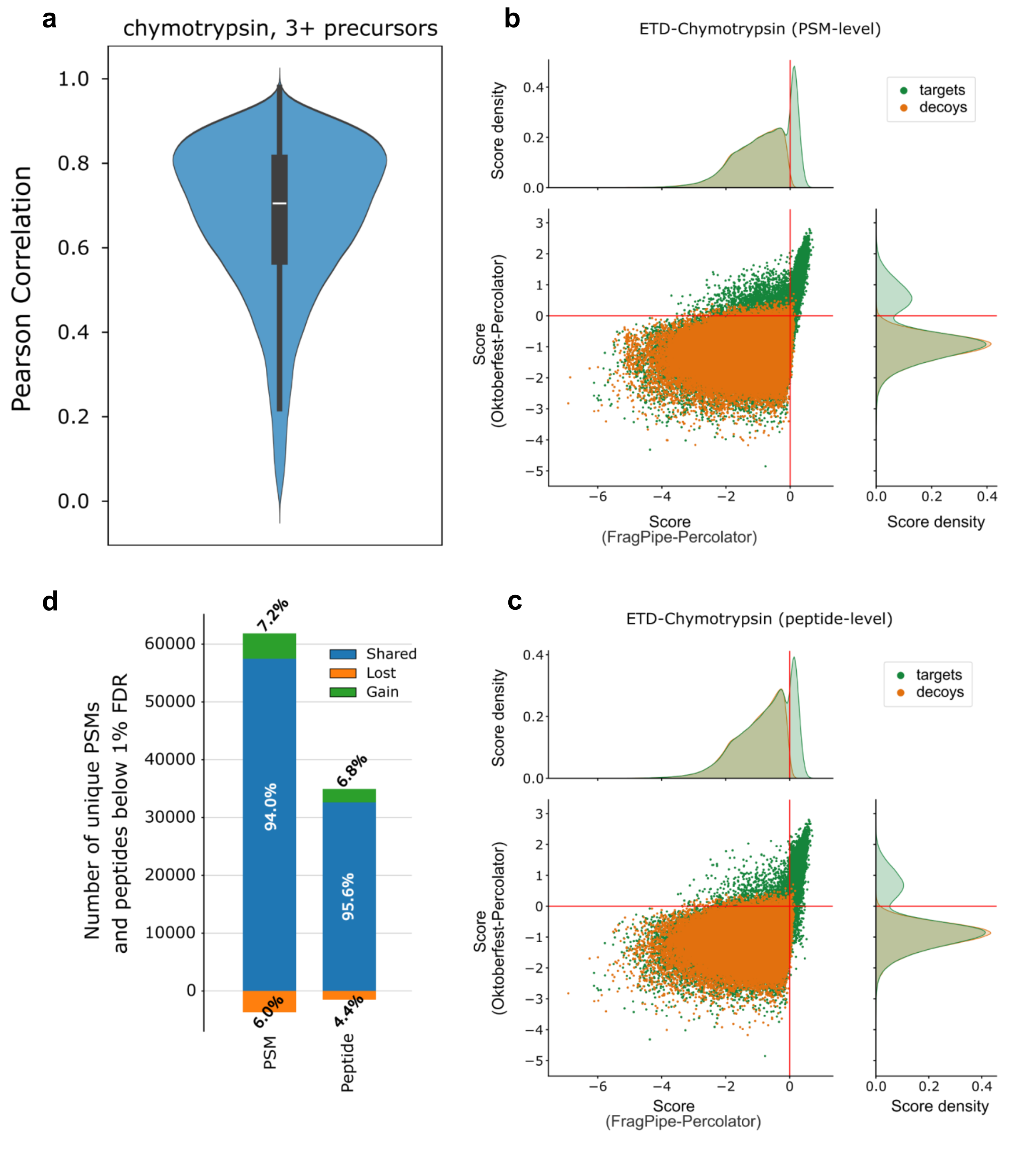

Supplementary Figure S25. Rescoring of the 3+ precursors from the publicly available ETD chymotrypsin dataset (Ref. [77]). a, Pearson correlation coefficient between experimental and predicted spectra of 3+ precursors from the ETD chymotrypsin dataset (Ref. [77]). **b-c,** Correlation of Percolator scores for all target (green) and decoy (orange) triply-charged PSMs (**b**) and peptide (**c**) in ETD chymotrypsin data acquired from rescoring the MSFragger (top) and Oktoberfest (right) sets of scores plotted per enzyme. The red lines indicate the 1% FDR cutoffs in MSFragger and Oktoberfest score distributions. **d**, Numbers of shared (blue), gained (green), and lost (orange) triply-charged PSMs (left) and peptides (right) identified at 1% FDR using Oktoberfest compared to the original MSFragger searches (without MSBooster) in the ETD chymotrypsin data. The rescoring was carried out using ETciD Prosit model.
