# Supplementary material for: Integration of alternative fragmentation techniques into standard LC-MS workflows using a single deep learning model enhances proteome coverage
Source: Nat Methods. 2026 Mar 23;23(4):805–14. doi: 10.1038/s41592-026-03042-9 (PMC13076210; doi:10.1038/s41592-026-03042-9)
Supplement: Supplementary file 2 — Reporting Summary [file 41592_2026_3042_MOESM2_ESM.pdf]

nature portfolio

Corresponding author(s): Shabaz Mohammed
Mathias Wilhelm

Last updated by author(s): 18/12/25

Reporting Summary

Nature Portfolio wishes to improve the reproducibility of the work that we publish. This form provides structure for consistency and transparency in reporting. For further information on Nature Portfolio policies, see our Editorial Policies and the Editorial Policy Checklist.

Statistics

For all statistical analyses, confirm that the following items are present in the figure legend, table legend, main text, or Methods section.

n/a	Confirmed	
☐	☒	The exact sample size (*n*) for each experimental group/condition, given as a discrete number and unit of measurement
☐	☒	A statement on whether measurements were taken from distinct samples or whether the same sample was measured repeatedly
☒	☐	The statistical test(s) used AND whether they are one- or two-sided<br>*Only common tests should be described solely by name; describe more complex techniques in the Methods section.*
☒	☐	A description of all covariates tested
☒	☐	A description of any assumptions or corrections, such as tests of normality and adjustment for multiple comparisons
☒	☐	A full description of the statistical parameters including central tendency (e.g. means) or other basic estimates (e.g. regression coefficient) AND variation (e.g. standard deviation) or associated estimates of uncertainty (e.g. confidence intervals)
☒	☐	For null hypothesis testing, the test statistic (e.g. *F*, *t*, *r*) with confidence intervals, effect sizes, degrees of freedom and *P* value noted<br>*Give P values as exact values whenever suitable.*
☒	☐	For Bayesian analysis, information on the choice of priors and Markov chain Monte Carlo settings
☒	☐	For hierarchical and complex designs, identification of the appropriate level for tests and full reporting of outcomes
☐	☒	Estimates of effect sizes (e.g. Cohen's *d*, Pearson's *r*), indicating how they were calculated

Our web collection on statistics for biologists contains articles on many of the points above.

Software and code

Policy information about availability of computer code

Data collection	LCMS spectra were collected on a custom Orbitrap Exploris-Omnitrap instrument and on an Orbitrap Ascend Tribrid instrument. After acquisition, data were processed in MSFragger (v4.1) within FragPipe (v22.0 or v23.0).
Data analysis	The "MultiFrag" model presented in the article was coded in Python using Pytorch v2.6.0 library. The training code for this model is available on Github (https://github.com/wilhelm-lab/Prosit_multifrag) and as a Koina instance at https://koina.proteomicsdb.org/. The rescoring of the initial MSFragger searches was performed in Oktoberfest (https://github.com/wilhelm-lab/oktoberfest) and MSBooster (v1.3.10) via FragPipe (v23.0). FDR estimations were done using Percolator (v3.6.5 and v3.7.1) and Philosopher (v5.1.1). Downstream data processing was done using Python 3.10. Python 3.10, R (4.2.2), Inkscape (v1.4) were used to prepare figures.

For manuscripts utilizing custom algorithms or software that are central to the research but not yet described in published literature, software must be made available to editors and reviewers. We strongly encourage code deposition in a community repository (e.g. GitHub). See the Nature Portfolio guidelines for submitting code & software for further information.

Data

Policy information about availability of data

All manuscripts must include a data availability statement. This statement should provide the following information, where applicable:

- Accession codes, unique identifiers, or web links for publicly available datasets
- A description of any restrictions on data availability
- For clinical datasets or third party data, please ensure that the statement adheres to our policy

The LCMS proteomics data, database MSFragger searches and results of data rescoring were deposited to the ProteomeXchange Consortium via the PRIDE partner repository with the dataset identifier PXD065289. Model weights, training, test, and holdout data sets were deposited to Zenodo (doi:10.5281/zenodo.15755223 and https://zenodo.org/records/15064340)

Research involving human participants, their data, or biological material

Policy information about studies with human participants or human data. See also policy information about sex, gender (identity/presentation), and sexual orientation and race, ethnicity and racism.

Reporting on sex and gender	n/a
Reporting on race, ethnicity, or other socially relevant groupings	n/a
Population characteristics	n/a
Recruitment	n/a
Ethics oversight	n/a

Note that full information on the approval of the study protocol must also be provided in the manuscript.

Field-specific reporting

Please select the one below that is the best fit for your research. If you are not sure, read the appropriate sections before making your selection.

☒ Life sciences ☐ Behavioural & social sciences ☐ Ecological, evolutionary & environmental sciences

For a reference copy of the document with all sections, see nature.com/documents/nr-reporting-summary-flat.pdf

Life sciences study design

All studies must disclose on these points even when the disclosure is negative.

Sample size	n/a
Data exclusions	n/a
Replication	n/a
Randomization	n/a
Blinding	n/a

Reporting for specific materials, systems and methods

We require information from authors about some types of materials, experimental systems and methods used in many studies. Here, indicate whether each material, system or method listed is relevant to your study. If you are not sure if a list item applies to your research, read the appropriate section before selecting a response.

Materials & experimental systems

n/a	Involved in the study
☒	☐ Antibodies
☐	☒ Eukaryotic cell lines
☒	☐ Palaeontology and archaeology
☒	☐ Animals and other organisms
☒	☐ Clinical data
☒	☐ Dual use research of concern
☐	☒ Plants

Methods

n/a	Involved in the study
☒	☐ ChIP-seq
☒	☐ Flow cytometry
☒	☐ MRI-based neuroimaging

Eukaryotic cell lines

Policy information about cell lines and Sex and Gender in Research

Cell line source(s)	Expi293F Cells were purchased from Thermo Fisher Scientific. Expi293F human cells are derived from the 293 cell line and are a core component of the Expi293 Expression System.
Authentication	n/a; no further authentication was performed in-house.
Mycoplasma contamination	The cell line was specified negative for mycoplasma by the vendor in a mycoplasma qPCR assay
Commonly misidentified lines (See ICLAC register)	No commonly misidentified cell lines were used in this study.

Dual use research of concern

Policy information about dual use research of concern

Hazards

Could the accidental, deliberate or reckless misuse of agents or technologies generated in the work, or the application of information presented in the manuscript, pose a threat to:

No	Yes
☒	☐ Public health
☒	☐ National security
☒	☐ Crops and/or livestock
☒	☐ Ecosystems
☒	☐ Any other significant area

Experiments of concern

Does the work involve any of these experiments of concern:

No	Yes
☒	☐ Demonstrate how to render a vaccine ineffective
☒	☐ Confer resistance to therapeutically useful antibiotics or antiviral agents
☒	☐ Enhance the virulence of a pathogen or render a nonpathogen virulent
☒	☐ Increase transmissibility of a pathogen
☒	☐ Alter the host range of a pathogen
☒	☐ Enable evasion of diagnostic/detection modalities
☒	☐ Enable the weaponization of a biological agent or toxin
☒	☐ Any other potentially harmful combination of experiments and agents

Plants

Seed stocks	Arabidopsis thaliana Col-0 seedling material was gifted by Cara Griffiths from Rothamsted Research
Novel plant genotypes	n/a
Authentication	not performed
